# Thermal Damage of Serpentine Concrete Under Microwave Volumetric Versus Muffle Furnace Surface Heating

**DOI:** 10.3390/ma19194139

**Published:** 2026-09-28

**Authors:** Qiongfang Wu, Zehua Liu, Zhenfu Chen, Dan Wu

**Affiliations:** 1 School of Resources Environment and Safety Engineering, University of South China, Hengyang 421001, China; 2009000110@usc.edu.cn; 2 School of Civil Engineering, University of South China, Hengyang 421001, China; 3 China Nuclear Industry Key Laboratory of High-Performance Concrete, University of South China, Hengyang 421001, China; 4 Hunan Provincial Key Laboratory of High-Performance Special Concrete, University of South China, Hengyang 421001, China

**Keywords:** serpentine concrete, microwave heating, thermal damage, temperature gradient, correction factor

## Abstract

Serpentine concrete is a key shielding material in nuclear engineering, and its thermal damage behaviour under irradiation-induced self-heating is closely related to structural safety. This study compares microwave volumetric heating (MW), which simulates internal heat generation, with muffle furnace surface heating (MF), which represents conventional external heating, at temperatures from 150 to 800 °C. The two heating mechanisms produced opposite temperature gradients: MW generated an inward-to-outward gradient, while MF produced a top-to-bottom gradient. These contrasting gradients led to distinct damage pathways—inside-out cracking versus top-down spalling—and different damage progression rates. In the critical dehydration range of 300–450 °C, the mass loss increment under volumetric heating (7.40%) was 3.2 times that under surface heating (2.29%). At 450 °C, the strength retention of the MW group (20.4%) was substantially lower than that of the MF group (77.7%). A heating-mechanism correction factor κ ≈ 0.25 is proposed to adjust surface-heating-based strength predictions to volumetric heating conditions. A comprehensive damage index D, integrating mass loss, crack development, and mechanical degradation, showed that severe damage under volumetric heating occurs approximately 200 °C earlier than under surface heating. These findings provide a quantitative framework for assessing thermal damage in concrete subjected to internal heat generation, with implications for nuclear safety assessments.

## 1. Introduction

Nuclear shielding concrete is widely used in biological shielding structures of nuclear reactors, spent fuel storage casks, and radioactive waste management facilities because it provides structural support while effectively attenuating neutron and gamma radiation [1,2]. Among the various shielding aggregates, serpentine has been widely adopted because of its high chemically bound water content (approximately 10–15 wt.%), which provides abundant hydrogen for efficient neutron moderation while maintaining relatively good thermal stability before significant dehydroxylation occurs [2,3,4]. As temperature increases, progressive dehydroxylation induces changes in the crystal structure of serpentine, accompanied by phase transformation, pore evolution, and microcrack development, which progressively deteriorate the mechanical properties and radiation shielding performance of the concrete [4,5]. Understanding this degradation behaviour under elevated temperatures is therefore essential for evaluating the long-term safety of nuclear shielding structures.

Nuclear shielding concrete is exposed to different thermal environments during the operation of nuclear facilities [6,7]. During normal reactor operation, energy deposited by neutron and gamma irradiation gives rise to internal volumetric heat generation throughout the concrete [6,7]. In contrast, under external thermal loading, heat is transferred from the exposed surface towards the interior through thermal conduction, representing a fundamentally different heat-transfer mechanism from irradiation-induced volumetric heating [8,9]. Consequently, these different heat-input modes produce distinct temperature fields within concrete, thereby influencing its thermal response and damage characteristics [8,9]. Moisture migration, pore pressure build-up, and thermally induced cracking under elevated-temperature exposure have been widely reported [9,10,11]. These observations highlight the importance of temperature-field evolution in understanding the thermal response of concrete, and underscore the need to clarify how different heat-input modes affect this evolution.

Furnace heating has been widely used to investigate the thermal response of concrete under external heating. Studies using in situ neutron tomography [10], heating rate analysis [12], and systematic reviews of spalling mechanisms [13] and high-temperature degradation [14] have advanced the understanding of moisture transport, pore-pressure development, and material deterioration in externally heated concrete. These findings provide an important basis for investigating thermal damage induced by surface heating.

Microwave heating differs fundamentally from conventional furnace heating in that thermal energy is generated directly within the material through dielectric loss, rather than transferred inward from the external surface. Owing to this volumetric heating characteristic, microwave technology has been extensively investigated in cement and concrete engineering for material processing, thermal treatment, recycling, and rehabilitation [15,16,17]. Recent studies have examined microwave-induced temperature-field evolution [18], aggregate-dependent heating efficiency [19], electromagnetic–thermal coupling [20], and the distinct damage patterns compared with conventional heating [21,22]. Collectively, these studies demonstrate that microwave heating provides an effective laboratory approach for generating volumetric heat input.

It should be noted that microwave heating is not a perfect analogue of irradiation-induced volumetric heating. Microwave heating depends on the dielectric properties of the material and the applied frequency, and the heating efficiency changes as dehydration progresses [18,19,20]. In contrast, energy deposition from gamma rays and neutrons is generally more spatially uniform and does not depend on the dielectric loss associated with dehydration [6,7]. These differences should be considered when interpreting the temperature-field results. Nevertheless, both heating modes generate heat volumetrically within the material, making microwave heating a reasonable laboratory approach for simulating the volumetric heat-input mode [15,16,17,22].

For serpentine-based radiation-shielding concrete, neutron-shielding performance is closely related to the hydrogen-bearing water in serpentine aggregates. Previous studies have shown that serpentine concrete provides better fast-neutron shielding than ordinary concrete [1], that antigorite dehydrates and transforms at elevated temperature and high pressure [4], and that increasing temperature reduces mass, mechanical properties, and shielding performance [5,21]. Overall, heating induces bound-water loss, phase transformation, and microstructural damage, resulting in gradual deterioration of mechanical and shielding properties.

The thermal response of radiation-shielding concrete depends not only on heating temperature but also on the mode of heat input. Furnace heating transfers heat from the surface inward, whereas microwave heating generates heat internally through dielectric loss [16,22]. Wei et al. directly compared the two methods and observed clear differences in heating rate, temperature development, crack formation, and material damage [22]. Bai et al. further demonstrated that temperature gradients significantly affect the mechanical properties and damage evolution of concrete [23]. However, direct comparisons between surface and volumetric heating remain limited, and it remains unclear whether their different temperature-gradient directions lead to different moisture migration, crack evolution, and thermal damage processes in serpentine-based concrete. A recent review noted that existing studies on serpentine concrete have mainly focused on mechanical strength, with limited attention given to damage evolution and quantitative analysis [24]. Although quantitative methods integrating ultrasonic pulse and acoustic-emission parameters have been developed for evaluating thermal damage in conventional concrete [25], their applicability to serpentine-based concrete under different heat-input modes remains unclear.

In addition, current standardized testing procedures for evaluating thermal damage in concrete—including those developed by RILEM technical committees and national standards organizations—are predominantly based on surface heating protocols (e.g., furnace heating). Whether the damage characteristics observed under such surface-heating conditions are representative of those induced by internal volumetric heating remains an open question. Without a clear understanding of how different heating mechanisms affect damage evolution, safety assessments based on conventional furnace tests may be incomplete for structures subjected to internal heat generation.

To address these knowledge gaps, this study employs muffle-furnace and microwave heating to represent two distinct heat-input paths. Muffle-furnace heating transfers heat from the specimen surface to the interior, whereas microwave heating generates volumetric heat within the material through dielectric loss, providing a laboratory analogue of internal energy deposition. Five target temperatures (150, 300, 450, 600, and 800 °C) are selected to cover the potential thermal conditions of serpentine-based concrete. Its service temperature near reactor cores is generally below 300 °C but may exceed 350 °C under abnormal heat transfer or reactor-system faults, while Generation IV nuclear systems may involve core temperatures of 500–800 °C [24,26]. The same five temperatures are applied to both heating methods to compare the effects of surface and volumetric heat input on temperature evolution, mass loss, crack development, and residual compressive strength. The objectives are: (1) to clarify how opposite temperature-gradient directions affect crack initiation and propagation; (2) to reveal the relationship between temperature-field evolution, dehydration, and damage development under different heating modes; (3) to establish a quantitative relationship between thermal response and strength degradation, including a heating-mechanism correction factor; and (4) to develop a comprehensive damage indicator that integrates mass loss, crack characteristics, and mechanical deterioration, providing a methodology applicable to the thermal damage assessment of building materials.

## 2. Methods

### 2.1. Raw Materials

Ordinary Portland cement (PO 42.5, Conch Cement Co., Ltd., Wuhu, China) was used, conforming to GB 175–2023 [27]. Serpentine aggregates were sourced from Hanzhong, Shaanxi Province, China. XRD analysis (Figure 1a) identified chrysotile and lizardite as the main mineral phases. The chemical composition of the serpentine aggregate is given in Table 1. Continuously graded coarse aggregate (5–20 mm) and serpentine sand (0–5 mm, fineness modulus 2.79) were used, with both satisfying GB/T 14685–2022 [28], GB/T 14684–2022 [29], GB/T 34008–2017 [30], and JGJ 52–2006 [31].

Thermogravimetric analysis (TGA/DSC1/1100SF, 10 °C/min from room temperature to 800 °C) was performed on serpentine powder (Figure 1b). The main mass loss (11.68%) occurred between 540 and 700 °C, corresponding to dehydroxylation of serpentine minerals [32]. Based on the TG mass loss, the chemically bound water content of the aggregate was estimated as approximately 12.51%.

The concrete mix proportion (water–cement ratio 0.51) was 441 kg/m^3^ cement, 225 kg/m^3^ water, 626 kg/m^3^ serpentine sand (<5 mm), and 1027 kg/m^3^ serpentine coarse aggregate (5–20 mm), following NB/T 20378–2016 [33].

### 2.2. Preparation and Grouping of Test Specimens

Cubic specimens (100 × 100 × 100 mm^3^) were prepared according to GB/T 50081–2019 [34]. After casting, all specimens remained in the moulds for 24 h, followed by demoulding and curing at 20 ± 2 °C and ≥ 95% relative humidity for 28 days.

Two configurations were adopted: intact specimens for macroscopic thermal response evaluation, and sectioned specimens for internal temperature field measurement and crack characterization.

A total of 105 specimens were prepared: 50 intact, 50 sectioned, and 5 room-temperature controls. Intact and sectioned specimens were equally divided into microwave (MW) and muffle furnace (MF) heating groups, with five target temperatures (150, 300, 450, 600, and 800 °C) and five replicates per condition. The experimental grouping and measurements are summarized in Table 2.

After curing, the intact specimens were oven-dried at 105 ± 5 °C for 24 h to constant mass, cooled to room temperature, and weighed for initial mass ($m_{0}$). For sectioned specimens, the cured cubes were cut along the centreline under continuous water cooling, and the mating surfaces were ground smooth and marked for reassembly. The sectioned specimens were then oven-dried at 105 ± 5 °C for 24 h to constant mass, cooled to room temperature, reassembled according to the markings, and secured with heat-resistant clamps.

Five additional intact specimens were retained as the room-temperature control group for determining initial compressive strength.

### 2.3. Heat Treatment Equipment and Methods

A JYS-6 microwave sintering furnace (6 kW, 2450 ± 50 MHz) was used for microwave heating (MW group). An SX2-36-13 silicon carbon rod box-type muffle furnace (36 kW) was used for conventional surface heating (MF group). For the MF group, the heating rate was controlled by a temperature program at 10 °C/min, and the specimen temperature closely followed the furnace temperature. For the MW group, the microwave power was adjusted so that the chamber temperature increased at approximately 10 °C/min. This chamber temperature can be approximately regarded as the surface temperature of the specimen. However, the internal temperature of the specimen was governed by volumetric microwave absorption and was considerably higher than the surface temperature; its heating rate could not be directly controlled by the temperature program. This difference in heating control should be considered when comparing the thermal damage between the two groups. Both groups were held at the target temperature for 30 min and then naturally cooled to room temperature.

### 2.4. Temperature Field Measurement

A Fotic 346 handheld thermal imager (768 × 576 pixels, thermal sensitivity <30 mK, accuracy ±2 °C) was used for temperature recording [35]. External surface temperature fields of intact specimens were recorded immediately after opening the furnace door. For sectioned specimens, the two halves were rapidly separated inside the furnace, and the internal cross-section temperature field was recorded within 5–10 s. Although the furnace air temperature decreased rapidly after opening, the specimen temperature decreased much more slowly, and the internal cross-sectional temperature difference changed only slightly. This is attributed to the low thermal diffusivity of concrete, which limits the heat penetration depth to only a few millimetres within 10 s. Consequently, the internal temperature field beyond this thin surface layer remained essentially unaffected. All thermal images were analysed using AnalyzIR software [36].

Three parameters were extracted:

External surface temperature difference:

(1) $$\Delta T_{s}=T_{s}^{\max}-T_{s}^{\min}$$

Internal cross-section temperature difference:

(2) $$\Delta T_{I}=T_{I}^{\max}-T_{I}^{\min}$$

Thermal value (*TV*)

(3) $$TV=10\times \frac{\sigma_{T}}{T_{\mathrm{Avg}}}$$

where $T_{S}^{\max}$ and $T_{S}^{\min}$ are the maximum and minimum temperatures on the specimen surface; $T_{I}^{\max}$ and $T_{I}^{\min}$ are the maximum and minimum temperatures on the internal cross-section; $\sigma_{T}$ is the standard deviation and $T_{\mathrm{Avg}}$ is the average temperature of all pixels within the selected region.

### 2.5. Mass Loss and Residual Compressive Strength

Mass was measured before and after heat treatment using an electronic balance (0.01 g accuracy, 5 kg capacity). The mass loss rate was calculated as:

(4) $$\Delta m=(m_{0}-m_{T})/m_{0}\times 100\%$$

where $m_{0}$ is the initial mass after pretreatment and $m_{T}$ is the mass after heating and cooling. Results are reported as the average of five parallel measurements.

Residual compressive strength was determined following GB/T 50081–2019 [34], using a TYE-600E compression testing machine at a loading rate of 0.3 MPa/s. The strength retention rate was calculated as:

(5) $$R_{f}=f_{cu,T}/f_{cu,RT}\times 100\%$$

where $f_{cu,T}$ is the compressive strength after heat treatment and $f_{cu,RT}$ is the initial compressive strength of the control group. All quantitative results are expressed as the mean ± standard deviation of five replicate specimens (n = 5), unless otherwise stated. Error bars in all figures represent one standard deviation.

### 2.6. Analysis of Crack Characteristics

Observation surfaces were coated with white matte spray paint to enhance crack contrast. High-resolution digital images (4K) were acquired with a standard scale bar. Image processing was performed in MATLAB R2023b using grayscale conversion, contrast enhancement, median filtering, and Otsu thresholding for binarization [37,38,39]. The box-counting fractal dimension was calculated as:

(6) $$D_{b}=\lim_{\varepsilon \to 0}\frac{\log N(\varepsilon )}{\log(1/\varepsilon )}\backslash tag1$$

where ε is the grid side length (pixels)($\varepsilon =2^{k},\;k=4,5,\ldots ,11$) and N(ε) is the number of grid cells covering the crack pattern. $D_{b}$ was obtained from the slope of linear regression of logN(ε) vs. log(1/ε), with $R^{2}>0.95$ [40]. Macroscopic changes (colour variation, flaking, blistering, shrinkage) were also photographed and documented.

## 3. Results

### 3.1. Temperature Field

#### 3.1.1. Temperature Field Patterns

The temperature fields of the MW and MF groups were recorded using a thermal imager. Figure 2 and Figure 3 present the external surface and internal cross-section temperature fields at target temperatures of 300 °C and 800 °C, respectively. These two temperatures represent the onset of internal cracking and the final stage of severe damage. The patterns at other temperatures (150, 450, and 600 °C) follow the same trend, and their quantitative data are provided in Table 3 and Figure 4.

In the MW group, both the external surface and the internal cross-section exhibit concentric isotherms, with the highest temperature at the centre (Figure 2a,b and Figure 3a,b). However, the internal cross-section is significantly hotter than the external surface. For example, at 300 °C the internal maximum temperature is much higher than the external surface maximum (Table 3). The internal temperature difference $\Delta T_{I}$ exceeds the external surface temperature difference $\Delta T_{S}$ throughout the tested range. This reflects the volumetric heating mechanism: heat is generated inside the specimen while the surface loses heat to the surroundings, creating a steep inward-to-outward gradient.

In contrast, the MF group shows a layered pattern on both the external surface and the internal cross-section, with the highest temperature at the top and decreasing downward (Figure 2c,d and Figure 3c,d). The external and internal temperatures are relatively close to each other, and the internal temperature difference $\Delta T_{I}$ is much smaller than that in the MW group. For instance, at 450 °C the internal temperature difference $\Delta T_{I}$ in the MF group is only about one-fifth of that in the MW group (Table 3). This reflects the surface-heating mechanism: heat is supplied from the surface and transferred inward by conduction, resulting in a gentler temperature gradient. The layered pattern in the MF group, with the highest temperature at the top and the lowest at the bottom, is consistent with the furnace design: the heating elements are located on the top and side walls, while the bottom is not directly heated.

As the target temperature increases, the concentric pattern in the MW group and the layered pattern in the MF group persist, as shown in Figure 2 and Figure 3. The internal temperature difference $\Delta T_{I}$ in the MW group reaches its maximum at 600 °C and then decreases slightly at 800 °C, while in the MF group both internal and external temperature differences increase gradually up to 600 °C and then decrease slightly at 800 °C.

#### 3.1.2. Temperature Differences and Thermal Values

The temperature differences ($\Delta T_{S}$ and $\Delta T_{I}$) and thermal values (*TV*) provide a quantitative basis for comparing the temperature fields described above. Figure 4 presents these parameters as functions of target temperature, and Table 3 lists the corresponding numerical values.

In the MW group, the external surface $\Delta T_{S}$ decreased from 286.5 °C at 150 °C to 70.0 °C at 800 °C, while the internal cross-section $\Delta T_{I}$ first increased from 416.3 °C at 150 °C to 527.1 °C at 600 °C, then decreased to 425.8 °C at 800 °C. The internal *TV* decreased from 9.26 to 4.05, but remained 1.2–4.7 times the surface TV throughout the tested range, indicating a much stronger internal temperature gradient under volumetric heating. This behaviour is attributed to the pronounced standing-wave effect at low temperatures and the subsequent decrease in dielectric loss factor, which gradually homogenizes the temperature distribution [18,20].

In the MF group, the external surface $\Delta T_{S}$ varied within 41.9–98.2 °C, while the internal cross-section $\Delta T_{I}$ increased from 44.3 °C to 110.7 °C at 600 °C, then decreased slightly to 98.6 °C at 800 °C. The internal-to-external TV ratio remained close to unity (1.02–1.36), indicating a gentle and fairly uniform temperature field under surface heating.

Overall, the MW group consistently shows much larger internal temperature differences and thermal values than the MF group, as summarized in Figure 4 and Table 3.

The internal maximum temperature under MW heating was considerably higher than the nominal heating temperature (e.g., 563.3 °C at a nominal 150 °C; Table 3), owing to the volumetric heating mechanism described in Section 3.1.1. In contrast, the internal maximum temperature under MF heating remained close to the furnace temperature, consistent with the surface-heating mechanism.

The temperature-field characteristics described above are important for understanding the thermal damage behaviour of serpentine concrete. The opposite gradient directions between the two groups strongly influence where cracks initiate. In the MW group, the central hot zone promotes radial cracking from the interior outward, whereas in the MF group, the top-surface hot zone drives downward cracking. This is consistent with the crack patterns observed in this study.

In addition, the internal–external contrast within each group further explains the different damage modes. In the MW group, the much higher internal temperature and larger internal temperature difference accelerate dehydration and thermal stress in the interior, leading to inside-out cracking. In the MF group, the relatively uniform internal–external temperature field produces a gentler gradient and top-down cracking.

The evolution of the temperature field with increasing target temperature reflects the competition among volumetric heat generation, heat conduction, and thermal radiation. In the MW group, the surface temperature difference decreases as temperature rises because the dielectric loss factor decreases and heat conduction becomes more effective at homogenizing the temperature distribution. In contrast, the internal temperature difference first increases and then decreases slightly at the highest temperature, possibly owing to thermal radiation. In the MF group, both internal and external temperature differences increase gradually up to 600 °C and then decrease slightly at 800 °C.

The much larger internal temperature difference in the MW group is expected to accelerate dehydration and generate larger thermal stress. This provides a basis for the faster mass loss, more severe cracking, and greater strength loss observed in the MW group. These aspects are analysed in detail in Section 4.

Finally, these findings have implications for the safety assessment of nuclear shielding concrete. Because internal heat generation produces a very different temperature field from external heating, damage patterns and degradation rates cannot be directly inferred from conventional surface-heating tests. This supports the need for heating-mechanism-specific assessment methods, as proposed in this study.

### 3.2. Mass Loss

#### 3.2.1. Mass Loss Analysis

Mass loss increased with temperature in both groups (Figure 5, Table 4). In the 300–450 °C range, the mass loss increment of the MW group was 7.40%, compared with 2.29% for the MF group, a difference of 5.11 percentage points. The maximum difference between the two groups occurred at 450 °C, reaching 7.31 percentage points (12.43% for MW vs. 5.09% for MF). Above 450 °C, the growth rate of the MW group slowed, and the difference narrowed to 2.45 percentage points at 800 °C (16.38% for MW vs. 13.93% for MF). This indicates that the accelerating effect of volumetric heating is most significant in the critical dehydration range; at higher temperatures, the difference narrows as the dehydration reactions approach completion.

The greater mass loss in the MW group is mainly attributed to its higher internal temperature, which accelerates both the dehydration of the cement paste and the dehydroxylation of the serpentine aggregate. A minor contribution may also arise from the decomposition of carbonate minerals such as magnesite, as indicated by the slow mass-loss stage at 340–400 °C in the TG curve of the serpentine aggregate (Figure 1b). Other possible reactions, such as oxidation of minor constituents, would tend to increase the sample mass rather than decrease it and are therefore not considered a source of mass loss. Consequently, the mass loss data primarily reflect the release of water from C-S-H, portlandite, and serpentine minerals.

#### 3.2.2. Dehydration Process Based on Tg Curves

The TG curves in Figure 1b show that serpentine aggregate dehydration includes a slow stage at 340–400 °C (magnesite decomposition) and a rapid stage at 520–720 °C (serpentine dehydroxylation), with a total mass loss of 12.51%. In cement paste, C-S-H dehydration mainly occurs between 100 and 450 °C, and portlandite decomposes between 400 and 500 °C [41]. Based on the chemically bound water content of hydrated cement paste and the TG results of serpentine aggregate in Figure 1b, the overall water loss potential of serpentine concrete is estimated to be approximately 16–18%.

In the MF group, the internal temperature generally followed the nominal heating temperature. At 450 °C, the average internal temperature was 386.5 °C (Table 3), corresponding to the transition zone from slow to rapid dehydration. At 600 °C, it was 561.7 °C, corresponding to the peak of rapid aggregate dehydration. At 800 °C, the total mass loss reached 13.93%, close to the lower limit of the combined water loss potential.

In the MW group, the internal temperature was much higher than the nominal heating temperature. At 150 °C, the average internal temperature had already reached 449.8 °C, bypassing the slow dehydration stage and entering the rapid dehydration phase. At 300 °C, the maximum temperature at the centre reached 752.1 °C, exceeding the endpoint of the rapid dehydration stage; however, mass loss at this point was only 5.00%, indicating that the high-temperature zone remained limited in both volume and duration. When the heating temperature reached 450 °C, the average internal temperature increased to 701.3 °C, causing the high-temperature zone at the centre to expand rapidly. The dehydration reaction evolved from local nucleation to volume-wide propagation, and the rapid dehydration of the aggregate overlapped with that of the cement paste, jointly driving a sharp increase in mass loss.

It should be noted that the nominal heating temperature in the MW group does not represent the actual internal temperature. Theoretically, if the entire specimen were at 150 °C, only free water would evaporate; however, under microwave volumetric heating, the actual internal temperature is much higher, so decomposition occurs in the central high-temperature zone. Due to the large internal temperature gradient, the central high-temperature zone undergoes both C-S-H dehydration and portlandite dehydroxylation, whereas the outer region mainly undergoes free water evaporation. This is supported by the mass loss data in Table 4: at 150 °C, the MW group showed 2.84% mass loss, compared with only 1.25% for the MF group. The 2.84% loss at 150 °C indicates that only a limited volume of the specimen experienced decomposition, consistent with the steep temperature gradient.

In the MW group, dehydration progressed faster than in the MF group. At ≤300 °C, rapid vaporization of internal water in the central region formed local microcracks near the aggregate–paste interface. At 300–450 °C, microcracks gradually connected with increasing temperature, and water transport paths evolved from isolated cracks to interconnected channels. At 450–600 °C, crack propagation became dominant during dehydration, and crack growth and water release promoted each other, resulting in accelerated mass loss.

In the MF group, heat moved from the surface inward, and dehydration progressed layer by layer. Water escaped mainly by diffusion, leading to slower dehydration and more gradual crack development.

### 3.3. Crack Development

#### 3.3.1. Macroscopic Appearance Changes

Crack evolution followed the temperature gradient direction in each group (Figure 6). In Figure 6, the crack locations are outlined with red lines to aid identification.

In the MW group, cracks initiated at the centre and propagated radially outward. At 300 °C, isolated microcracks appeared around the centre of the internal cross-section (Figure 6c), while the external surface showed only slight whitening (Figure 6a). At 450 °C, the damaged zone expanded to approximately 50 mm in diameter, with the aggregate turning reddish-brown and radial cracks extending 15–30 mm outward; the external surface began to bulge. At 600 °C, the radial cracks interconnected into a through-crack network approximately 1–2 mm wide covering nearly the entire cross-section, accompanied by dense surface cracking and edge spalling. At 800 °C, a cavity approximately 2–3 cm in diameter formed at the centre, the surrounding framework fractured, and the specimen exhibited overall deformation and collapse.

In the MF group, cracks initiated at the upper surface and propagated downward. At 450 °C, fine cracks appeared at the top edges and corners of the external surface (Figure 6b); internal cracks extended downward from the upper region (Figure 6d). At 600 °C, longitudinal cracks developed on the external surface with noticeable top spalling; internal cracking was dense in the upper region (approximately 0.5 mm wide) but sparse in the middle and lower sections. At 800 °C, the upper part was severely fractured and spalled, while the middle and lower parts remained relatively intact.

The MW group thus exhibited an inside-out damage pattern, whereas the MF group exhibited a top-down progression. This contrast—cracks propagating from the centre outward versus from the surface downward—is consistent with the opposite temperature gradient directions observed in Section 3.1.

#### 3.3.2. Quantitative Analysis of Cracks

The crack identification and quantification workflow is illustrated in Figure 7, using the internal cross-section of the MW group at 450 °C as an example. The raw image was processed through multi-feature enhancement, multiscale fusion binarization, and noise reduction. The box-counting method was then applied to calculate the fractal dimension ($D_{b}$) from the slope of logN(ε) vs. log(1/ε), with $R^{2}>0.95$.

Figure 8, Figure 9, Figure 10 and Figure 11 present the binarized crack patterns for the external surfaces and internal cross-sections of both groups across all temperatures (150–800 °C). The quantitative results are summarized in Table 5 and plotted in Figure 12.

In the MW group, the internal cross-sections consistently exhibited higher $D_{b}$ values and larger crack areas than the external surfaces. The internal $D_{b}$ increased from 1.173 at 150 °C to 1.635 at 800 °C, while the external $D_{b}$ increased from 1.112 to 1.349. The internal crack area percentage rose from 0.11% to 8.71%, compared with 0.07% to 2.15% on the external surface. Maximum internal crack length and width grew from 5.32 mm and 0.45 mm at 150 °C to 98.44 mm and 4.93 mm at 800 °C. At 300 °C, early microcracks appeared at the centre of the internal cross-section before significant dehydration (mass loss ~5.00%), providing initial channels for vapour escape. Between 450 and 600 °C, the crack area increased rapidly, and cracks widened and interconnected into a through-crack network. Above 600 °C, internal cracks continued to grow while external surface cracks developed more slowly, consistent with the inside-out damage pattern.

In the MF group, the external surface $D_{b}$ increased from 1.073 at 150 °C to 1.392 at 800 °C, while the internal $D_{b}$ increased from 1.132 to 1.348. The external crack area rose from 0.03% to 4.04%, compared with 0.17% to 2.21% internally. Maximum external crack length and width reached 99.90 mm and 1.61 mm at 800 °C, while internal cracks reached only 42.73 mm and 2.14 mm. Up to 450 °C, cracks were limited; beyond this temperature, cracking accelerated on the external surface.

The MW group exhibited higher crack width and area percentage (max width 4.93 mm, area 8.71%), whereas MF cracks extended mainly along the temperature gradient (max length 99.90 mm) with limited width (1.61 mm). This contrast—cracks growing in width and forming a high-density network under volumetric heating versus propagating as narrow, elongated cracks under surface heating—reflects the different damage pathways identified in the macroscopic observations.

### 3.4. Residual Compressive Strength

Residual compressive strength decreased continuously with temperature in both groups (Figure 13, Table 6). In the 150–300 °C range, the strength retention rate of the MW group decreased by 22.4 percentage points (75.2% → 52.8%), while the MF group decreased by 9.3 percentage points (95.5% → 86.2%); the reduction in the MW group was approximately 2.4 times greater. In the critical dehydration range of 300–450 °C, the MW group decreased by 32.4 percentage points (52.8% → 20.4%), while the MF group decreased by only 8.5 percentage points (86.2% → 77.7%), representing a 3.8-fold difference. At 450 °C, the retention rate of the MW group had already fallen to 20.4%, whereas the MF group still retained 77.7% of its original strength—a difference of 57.3 percentage points. At 800 °C, the retention rates further decreased to 7.6% and 34.6%, respectively.

The strength degradation was closely correlated with dehydration and crack propagation. In the MW group, the mass loss increased sharply by 7.40% in the 300–450 °C range (Table 4), while the strength retention rate dropped by 32.4 percentage points over the same interval; the two trends were highly consistent. In the MF group, dehydration developed more gradually, and the decline in strength was also relatively gradual. When the internal $D_{b}$ exceeded 1.4 (450–600 °C range in the MW group), the strength retention rate decreased sharply to below 20%. In contrast, $D_{b}$ in the MF group remained below 1.4 throughout the heating process, and the strength decreased steadily without any abrupt drop or obvious inflection point. This suggests that the formation of a highly connected crack network was closely associated with the rapid loss of strength under volumetric heating conditions.

## 4. Discussion

### 4.1. Temperature Gradient Direction Determines Damage Initiation

The two heating mechanisms produced opposite temperature gradients. In the MW group, temperatures decreased from the centre outward (Figure 2 and Figure 3). In the MF group, temperatures decreased from the upper region downward. According to thermoelasticity theory, thermal stresses develop along temperature gradients, with tensile stress concentrated where the gradient is largest. In the MW group, tensile stress concentrated in the central region and promoted radial cracking from the interior outward (Section 3.3.1). In the MF group, tensile stress concentrated near the upper surface and drove cracking downward.

The crack patterns in Figure 6 and the quantitative data in Table 6 confirm that the crack initiation sites and propagation pathways followed the temperature gradient direction in each case. The different heating modes therefore resulted in distinct damage initiation regions and crack evolution pathways, as schematically illustrated in Figure 14.

### 4.2. Temperature Gradient Intensity Governs the Dehydration–Cracking Coupling

#### 4.2.1. Internal Temperature Difference and Dehydration Rate

The internal temperature difference ($\Delta T_{I}$) reflects the intensity of the thermal gradient within the specimen. The MW group consistently showed much larger $\Delta T_{i}$ than the MF group (Table 3, Figure 4). At 450 °C, $\Delta T_{i}$ in the MW group (474.5 °C) was roughly five times that in the MF group (96.8 °C) (Table 7).

The large internal temperature gradient in the MW group is sustained by the low thermal conductivity of concrete, typically 1.4–3.6 W/(m·K) [42]. This low conductivity slows heat transfer from the interior to the surface, so the heat generated by volumetric microwave absorption cannot be rapidly dissipated by conduction.

This stronger gradient pushed the central region of the MW specimens into the rapid dehydroxylation stage (520–720 °C, Figure 1b) much earlier. At a nominal 450 °C, the average internal temperature of the MW group reached 701.3 °C, while the MF group averaged only 386.5 °C (Table 3). As a result, in the 300–450 °C range, the mass loss increment of the MW group (7.40%) was 3.2 times that of the MF group (2.29%) (Table 4 and Table 7). The radial gradient thus accelerated dehydration significantly compared with the layered temperature field in the MF group.

#### 4.2.2. Temperature Gradient Intensity and Crack Development

Larger internal temperature differences were also closely linked to faster crack development. In the MW group, between 300 and 600 °C, $\Delta T_{I}$ rose from 454.1 °C to 527.1 °C, while the crack area increased from 2.19% to 6.91% (Table 8). The crack area increased as $\Delta T_{I}$ increased, showing that crack propagation accelerated with increasing temperature gradient. Between 600 and 800 °C, $\Delta T_{I}$ decreased slightly (527.1 °C → 425.8 °C), but the crack area continued to increase (6.91% → 7.74%), suggesting that at high temperatures, accumulated thermal damage and thermal softening also contributed to crack propagation.

In the MF group, $\Delta T_{I}$ remained below 111 °C throughout the heating process, and the crack area increased gradually without obvious stage changes, indicating that the milder temperature gradient under surface heating led to slower crack development.

#### 4.2.3. Synergistic Effect of Temperature Difference on Damage Evolution

The internal temperature $\Delta T_{I}$ difference is a key factor linking heat input to damage evolution. Larger gradients accelerate dehydration and increase thermal stress, which together accelerate crack growth and strength loss. In the MW group, where $\Delta T_{I}$ exceeded 400 °C, this effect was strong; in the MF group, with $\Delta T_{I}$ below 150 °C, the effect was small.

The distinct damage pathways are attributed to different dehydration–cracking coupling mechanisms. In the MW group, crack initiation preceded extensive dehydration: at 300 °C, early microcracks appeared at the centre of the internal cross-section, primarily along the aggregate–paste interface, before significant dehydration (mass loss 5.00%). These microcracks provided initial channels for water vapour escape. As temperature rose from 300 to 450 °C, they gradually linked into a continuous network, reducing diffusion resistance and accelerating further dehydration. The growing vapour pressure simultaneously drove further crack propagation. This formed a positive feedback loop (dehydration → vapour pressure → cracking → enhanced vapour transport → accelerated dehydration), as schematically illustrated in Figure 15a.

In the MF group, heat transferred from the surface inward, and dehydration progressed layer by layer. Water escaped mainly by diffusion, leading to slower dehydration and more gradual crack development. Cracks appeared after dehydration progressed in layers, and the limited crack width (maximum 1.61 mm) indicates that crack propagation was constrained by both heat penetration and material anisotropy. This sequential dehydration–shrinkage–cracking mechanism resulted in progressive damage without abrupt transitions, as schematically illustrated in Figure 15b.

The difference in crack initiation sites between the two groups can be further explained by the behaviour of the interfacial transition zone (ITZ). The ITZ is the weakest region in concrete due to its higher porosity and oriented portlandite. In both groups, cracks tended to follow the aggregate–paste interface (Figure 10 and Figure 11). In the MW group, the cement paste and serpentine aggregate have different dielectric properties, which can lead to non-uniform microwave absorption at the interface [43]. This non-uniform heating, together with the large internal temperature gradient, generates differential thermal stress at the interface [44]. The decomposition of portlandite further weakens the ITZ. As a result, interfacial microcracks were more pronounced and formed a dense network in the interior, consistent with the inside-out damage pattern. In the MF group, the gentler temperature gradient led to less pronounced interfacial cracking, mainly concentrated in the upper surface layer, consistent with the top-down cracking observed in Figure 6.

In addition to the interfacial effects discussed above, heating also causes shrinkage due to dehydration of the cement paste and dehydroxylation of the serpentine aggregate. In the MW group, the interior experiences higher temperatures and undergoes greater shrinkage than the outer region. Because the outer region constrains the interior shrinkage, tensile stress may develop in the interior. In the MF group, the surface undergoes greater shrinkage than the interior, and the surface is constrained by the interior, which may lead to tensile stress at the surface. These trends are consistent with the observed crack initiation sites, although thermal expansion, pore pressure, and material degradation may also contribute; their relative contributions cannot be quantified without further analysis.

It should also be noted that no post-test XRD or TG/DTG analysis was performed in this study to quantify the residual mineral phases, such as dehydrated C-S-H, unreacted portlandite, and anhydrous phases like forsterite or belite. Such analysis would provide a more complete understanding of the chemical mechanism and will be addressed in future work.

Given the absence of direct chemical evidence, a hypothesis based on previous studies is proposed here. Another potential chemical effect is alkali–thermal activation. The thermal decomposition of serpentine releases highly reactive amorphous silica. In the presence of alkalis from the cement, this silica may react to form alkali–silicate gels, which can cause expansion and microcracking. Similar behaviour has been reported for cement mortars with waste glass aggregate at high temperature [45]. In the MW group, macroscopic features such as surface bulging and central cavity formation were observed (Figure 6), which are consistent with an expansion-driven process. This mechanism is therefore proposed as a hypothesis and warrants further investigation.

The damage mechanisms discussed above could potentially be mitigated by fiber reinforcement. Previous studies have shown that fiber geometry, aspect ratio, and volume fraction significantly affect the crack-bridging capacity and ductility of cementitious composites after thermal damage [46]. In particular, steel fibers can bridge cracks and improve residual tensile behavior at high temperatures, while polypropylene fibers melt to create pathways for moisture escape, thereby reducing the risk of spalling [47]. Therefore, incorporating fibers into serpentine concrete may improve its residual mechanical performance under internal heat generation. This aspect was not included in the present study and will be addressed in future work.

### 4.3. Correction Factor for Volumetric Heating

#### 4.3.1. Heating Mechanism Correction Factor

To quantify the acceleration effect of volumetric heating relative to surface heating at the same nominal temperature, a correction factor κ(T) was defined as the ratio of the strength retention rates:

(7) $$\kappa (T)=R_{f,MW}/R_{f,MF}$$

where $R_{f,MW}$ and $R_{f,MF}$ are the strength retention rates of the MW and MF groups at the same temperature.

As shown in Table 9, κ(T) decreased continuously with temperature: 0.787 at 150 °C, 0.613 at 300 °C, 0.269 at 450 °C, 0.232 at 600 °C, and 0.220 at 800 °C. Above 450 °C, the values stabilized at approximately 0.22–0.27. For engineering applications, κ≈0.25 can be used as a practical approximation. When muffle furnace data are used to estimate residual strength under volumetric heating conditions, the measured strength retention can be corrected using this factor.

For practical engineering applications, the correction factor κ(T) can be simplified into the following piecewise function:

(8) $$\kappa (T)=\left\{\begin{array}{c} 0.7,T\leq 300\\ \mathrm{linear}\;\mathrm{interpolation},300<T<450\\ 0.25,T\geq 450 \end{array}\right.$$

When muffle furnace data are used to estimate the residual strength under volumetric heating conditions, the measured strength retention can be corrected using κ(T). This suggests that the correction factor provides a reasonable approximation for evaluating the acceleration effect caused by volumetric heating.

#### 4.3.2. Physical Origin of the Acceleration Effect

At 450 °C, the strength loss in the volumetric heating specimens was already roughly four times greater than that in the surface heating specimens. This difference arises from three factors. First, the much larger internal temperature gradient in the MW group ($\Delta T_{I}$ = 474.5 °C at 450 °C vs. 96.8 °C in the MF group) caused the central region to enter the rapid dehydration stage earlier. Second, the higher thermal value (*TV* = 6.77 vs. 2.50) promoted faster crack interconnection and damage growth. Third, the positive feedback between dehydration and cracking (Section 4.2.3) accelerated damage accumulation in the MW group, whereas the MF group exhibited a more gradual sequential deterioration process. These combined effects are captured by the correction factor κ≈0.25, which can be applied directly in design assessments and safety evaluations for environments with self-generated heat in nuclear facilities.

Microwave heating depends on the dielectric properties of the material and the applied frequency, and the heating efficiency changes as dehydration progresses. In this study, the serpentine concrete specimens exhibited a regular concentric temperature field under microwave heating, without pronounced local hot spots. This is attributed to the combined effect of the uniformly distributed water (both free water and chemically bound water in serpentine aggregate) that efficiently absorbs microwave energy, and the relatively low dielectric loss of the serpentine aggregate itself, which prevents localized overheating. Nevertheless, the internal temperature $\Delta T_{I}$ under microwave heating was considerably higher than the nominal heating temperature, whereas under muffle-furnace heating it remained close to the furnace temperature. Therefore, the temperature-field results reported here should be considered as a conservative case for volumetric heating.

### 4.4. Comprehensive Damage Index

#### 4.4.1. Definition of the Indicator

To provide a comparative evaluation of thermal damage, a dimensionless comprehensive damage index D∈[0,1] was defined by combining mass loss, internal crack development, and strength reduction:

(9) $$D=\frac{1}{3}(\frac{\Delta m}{\Delta m_{\max}}+\frac{A_{crack}}{A_{\max}}+\frac{100-R_{f}}{100})$$

where Δm is the mass loss rate (%), $A_{\mathrm{crack}}$ is the crack area percentage (%), $R_{f}$ is the strength retention rate (%), and the normalization values ($\Delta m_{\max}$, $A_{\max}$) correspond to the maximum measured values of the MW group at 800 °C. This index is intended to provide a relative description of damage evolution and facilitate comparison between different heating mechanisms.

#### 4.4.2. Temperature Evolution of the *D*-Value

The *D*-value increased continuously with temperature in both groups (Table 10, Figure 16), indicating progressive accumulation of thermal damage. The propagated uncertainty in *D* was approximately 0.01 for all conditions. At the same nominal temperature, the MW group consistently showed higher *D*-values than the MF group. At 450 °C, the MW group reached *D* = 0.51 ± 0.01 (moderate damage), while the MF group remained at *D* = 0.32 ± 0.01 (mild damage). At 600 °C, the MW group reached *D* = 0.69 ± 0.01 (severe damage), while the MF group reached the same level (*D* = 0.69 ± 0.01) only at 800 °C. Volumetric heating thus advanced the onset of severe damage by roughly 200 °C compared with surface heating.

#### 4.4.3. Damage Severity Classification

Based on the *D*-value, five severity levels are proposed in Table 11, together with the corresponding engineering recommendations: minor damage (*D* < 0.2), mild damage (0.2 ≤ *D* < 0.4), moderate damage (0.4 ≤ *D* < 0.6), severe damage (0.6 ≤ *D* < 0.8), and serious damage (*D* ≥ 0.8).

### 4.5. Engineering Criteria System

Table 12 integrates the main findings into a practical framework for assessing concrete exposed to internal heat generation. The framework uses the temperature gradient direction, internal temperature difference, crack characteristics, fractal dimension, correction factor κ, and the damage index *D* to guide engineering decisions. It provides a basis for adjusting predictions derived from conventional surface-heating tests to conditions relevant to irradiation-induced self-heating in nuclear facilities.

## 5. Conclusions

This study investigated the thermal damage behaviour of serpentine concrete under microwave volumetric heating (MW) and muffle furnace surface heating (MF) at temperatures ranging from 150 to 800 °C. The main conclusions are as follows:

(1) The two heating mechanisms produced fundamentally different temperature-gradient directions, which governed the crack initiation sites and propagation pathways. Microwave volumetric heating generated an inward-to-outward temperature gradient, causing cracks to initiate at the specimen centre and propagate radially outward. In contrast, muffle furnace surface heating produced a top-to-bottom temperature gradient, resulting in crack initiation at the upper surface and downward propagation. These contrasting damage pathways—inside-out versus top-down—confirm that the direction of the temperature gradient, rather than the temperature level alone, determines the spatial evolution of thermal damage.
(2) The internal temperature gradient intensity was identified as the key parameter controlling damage progression. At 450 °C, the internal temperature difference under volumetric heating reached 474.5 °C, approximately 4.9 times that under surface heating (96.8 °C). In the critical dehydration range of 300–450 °C, the mass loss increment under volumetric heating (7.40%) was 3.2 times greater than that under surface heating (2.29%). This accelerated dehydration, in turn, promoted faster crack development and strength degradation, demonstrating that thermal damage is governed not only by temperature level but also by the intensity of the internal thermal gradient.
(3) Distinct dehydration–cracking coupling mechanisms were observed under the two heating modes. Under volumetric heating, crack initiation preceded extensive dehydration, creating a positive feedback loop between cracking and dehydration that accelerated damage evolution. Under surface heating, dehydration preceded cracking in a layer-by-layer fashion, resulting in a more gradual deterioration process. This difference explains the accelerated damage progression under internal heat generation.
(4) A heating-mechanism correction factor *κ* ≈ 0.25 was established to quantify the acceleration effect of volumetric heating relative to surface heating for temperatures above 450 °C. This factor allows strength predictions derived from conventional surface-heating tests to be adjusted for volumetric heating conditions relevant to irradiation-induced self-heating in nuclear shielding concrete.
(5) A comprehensive damage index *D*, integrating mass loss, crack characteristics, and strength reduction, was proposed to evaluate the overall thermal damage. Based on *D*-values, severe damage (*D* ≈ 0.69) occurred at 600 °C under volumetric heating, whereas the same level of damage was not reached until 800 °C under surface heating—a temperature offset of approximately 200 °C. This offset highlights the importance of accounting for heating mechanisms in thermal safety assessments of nuclear shielding concrete.

In summary, the proposed framework—including the temperature-gradient direction criterion, the correction factor *κ*, and the comprehensive damage index *D*—provides a practical basis for assessing the thermal damage of serpentine-based radiation-shielding concrete subjected to internal heat generation, and for correcting predictions derived from conventional surface-heating tests.

## Figures and Tables

**Figure 1:**
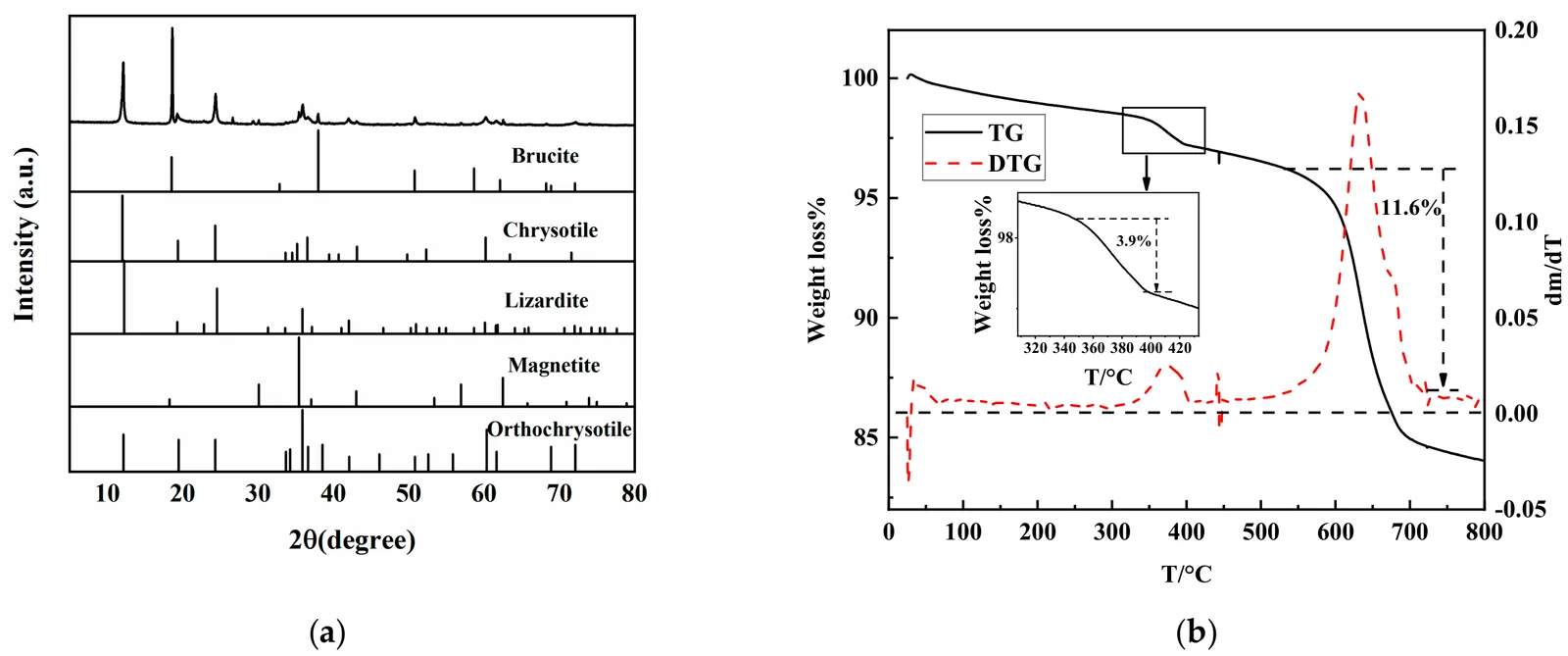
Serpentine aggregate characterization: (**a**) XRD pattern; (**b**) TG-DTG curves.

**Figure 2:**
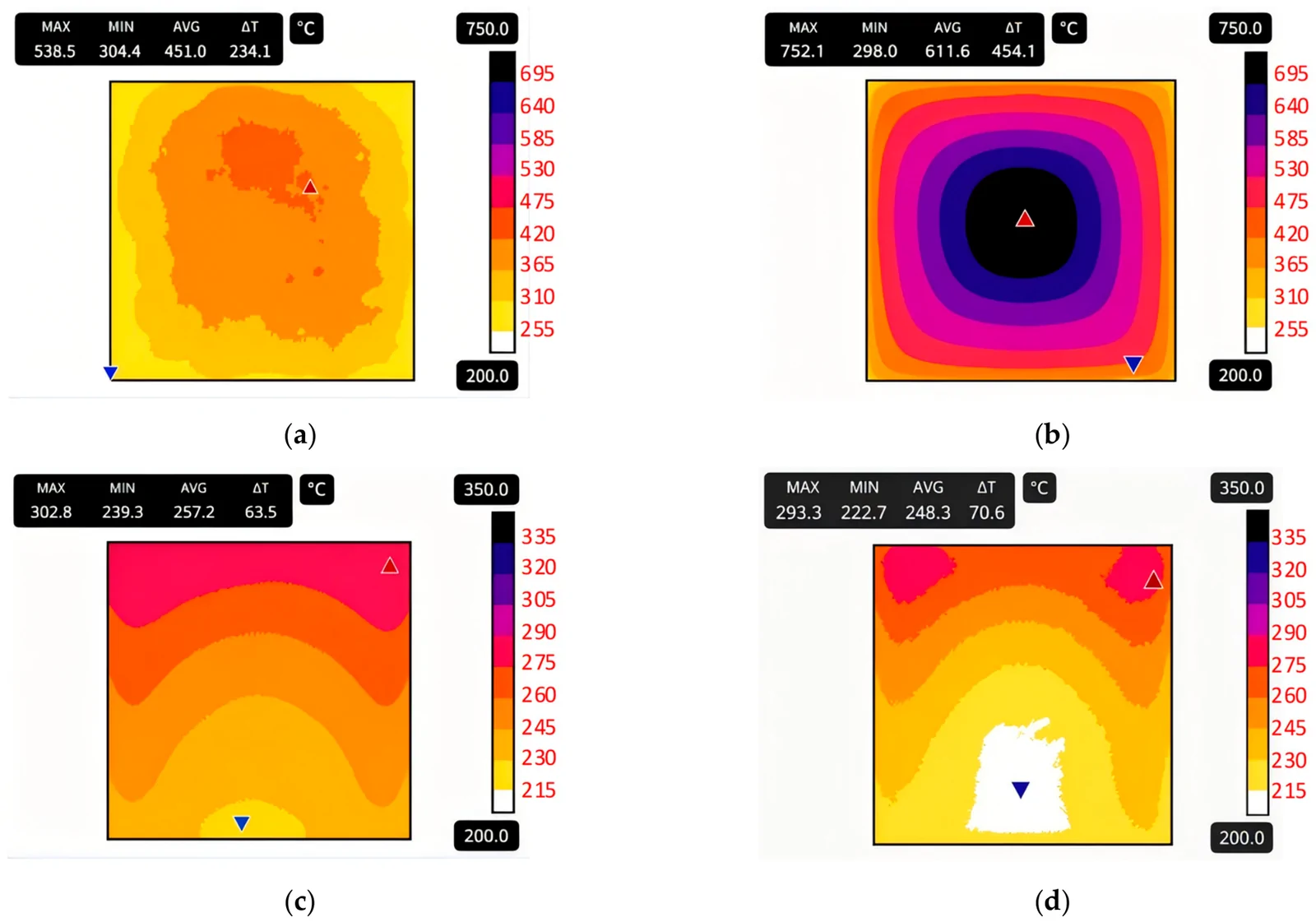
Comparison of temperature fields at 300 °C. (**a**) MW external surface temperature field; (**b**) MW internal cross-section temperature field; (**c**) MF external surface temperature field; (**d**) MF internal cross-section temperature field.

**Figure 3:**
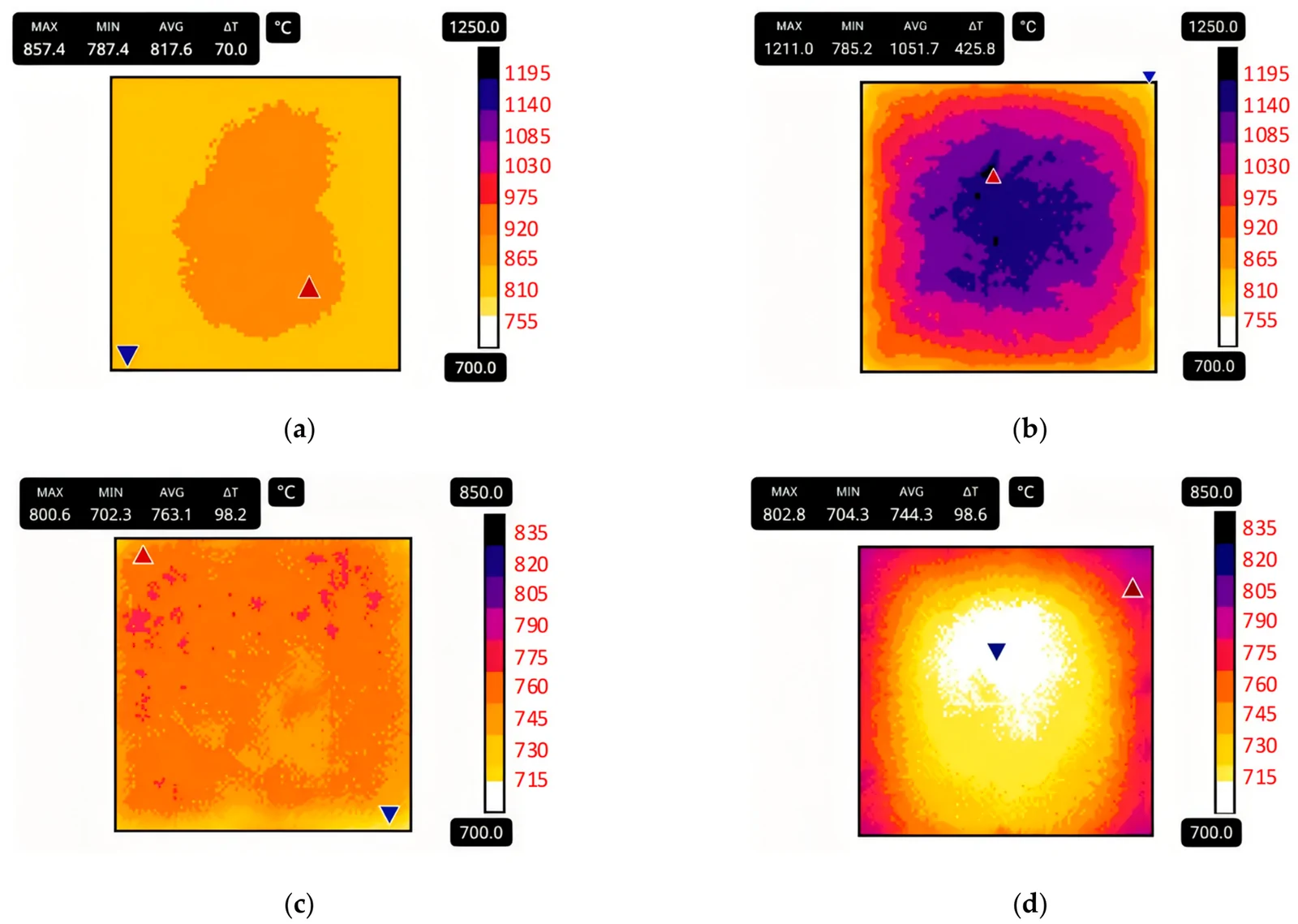
Comparison of temperature fields at 800 °C. (**a**) MW external surface temperature field; (**b**) MW internal cross-section temperature field; (**c**) MF external surface temperature field; (**d**) MF internal cross-section temperature field. The red upward-pointing arrow indicates the location of the maximum temperature, and the blue downward-pointing arrow indicates the location of the minimum temperature.

**Figure 4:**
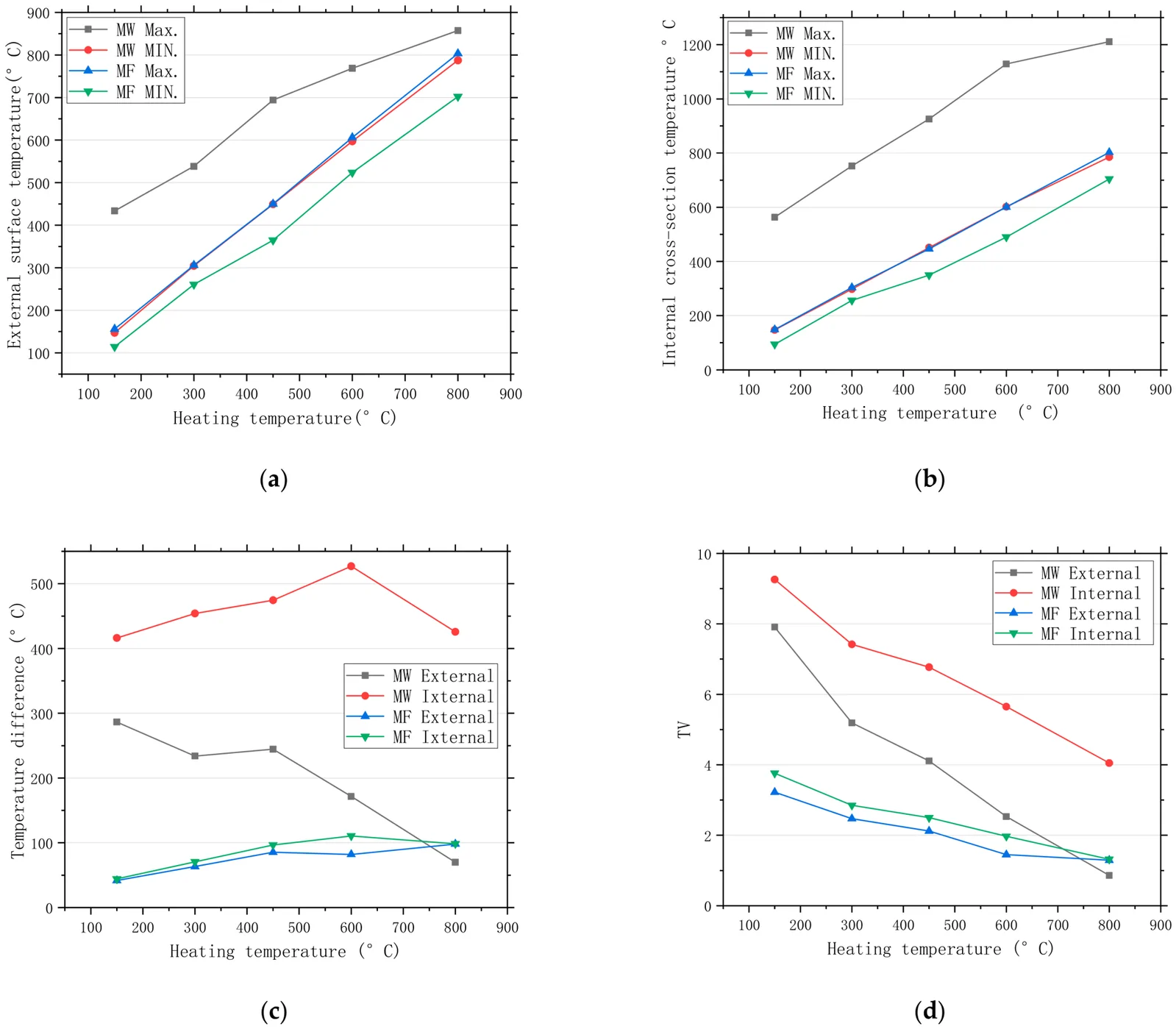
Variations in characteristic temperature differences and thermal values with heating temperature. (**a**) Maximum and minimum surface temperatures versus heating temperature; (**b**) maximum and minimum internal cross-section temperatures versus heating temperature; (**c**) temperature difference versus heating temperature; (**d**) thermal value (TV) versus heating temperature.

**Figure 5:**
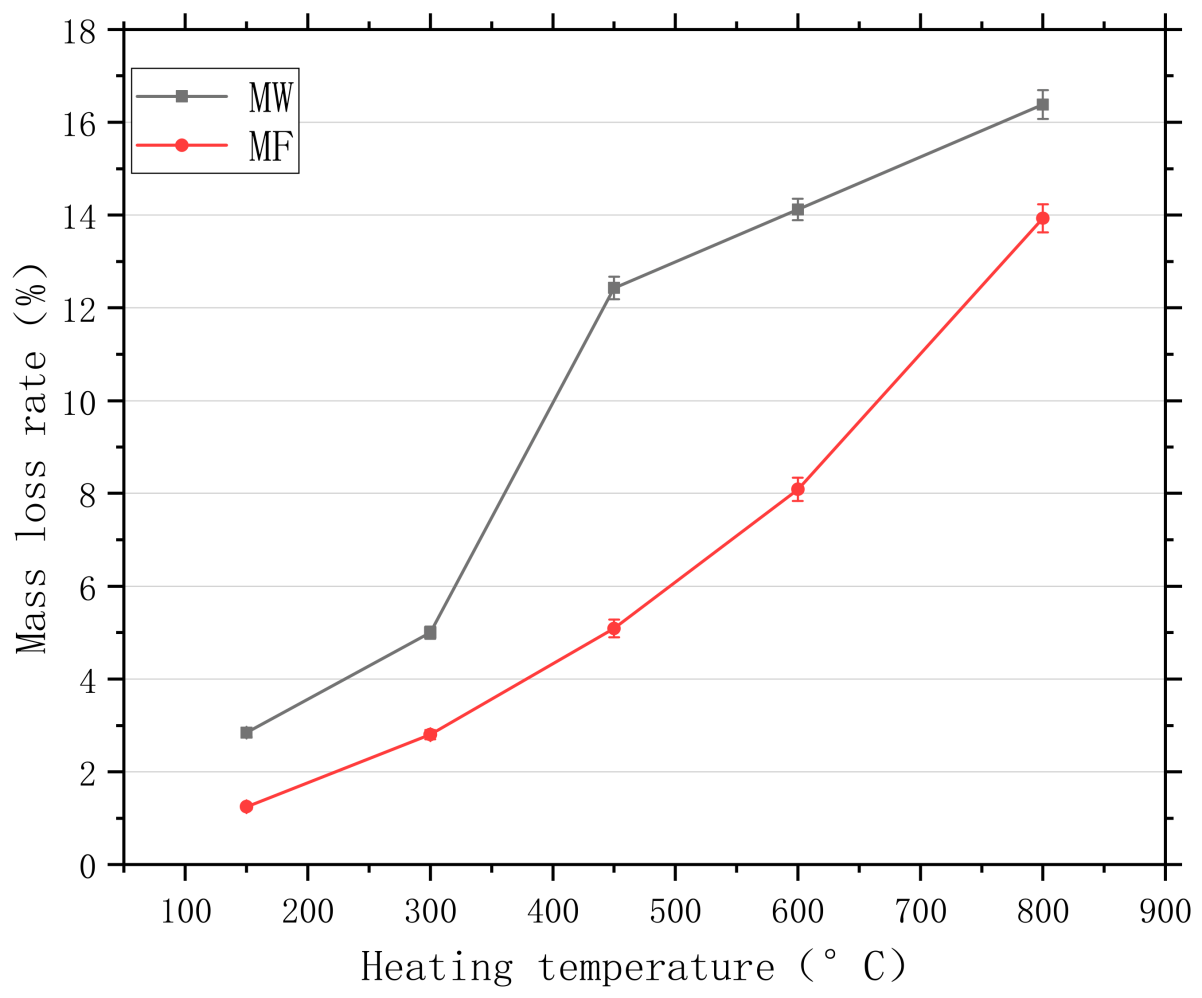
Variation in mass loss rate with heating temperature.

**Figure 6:**
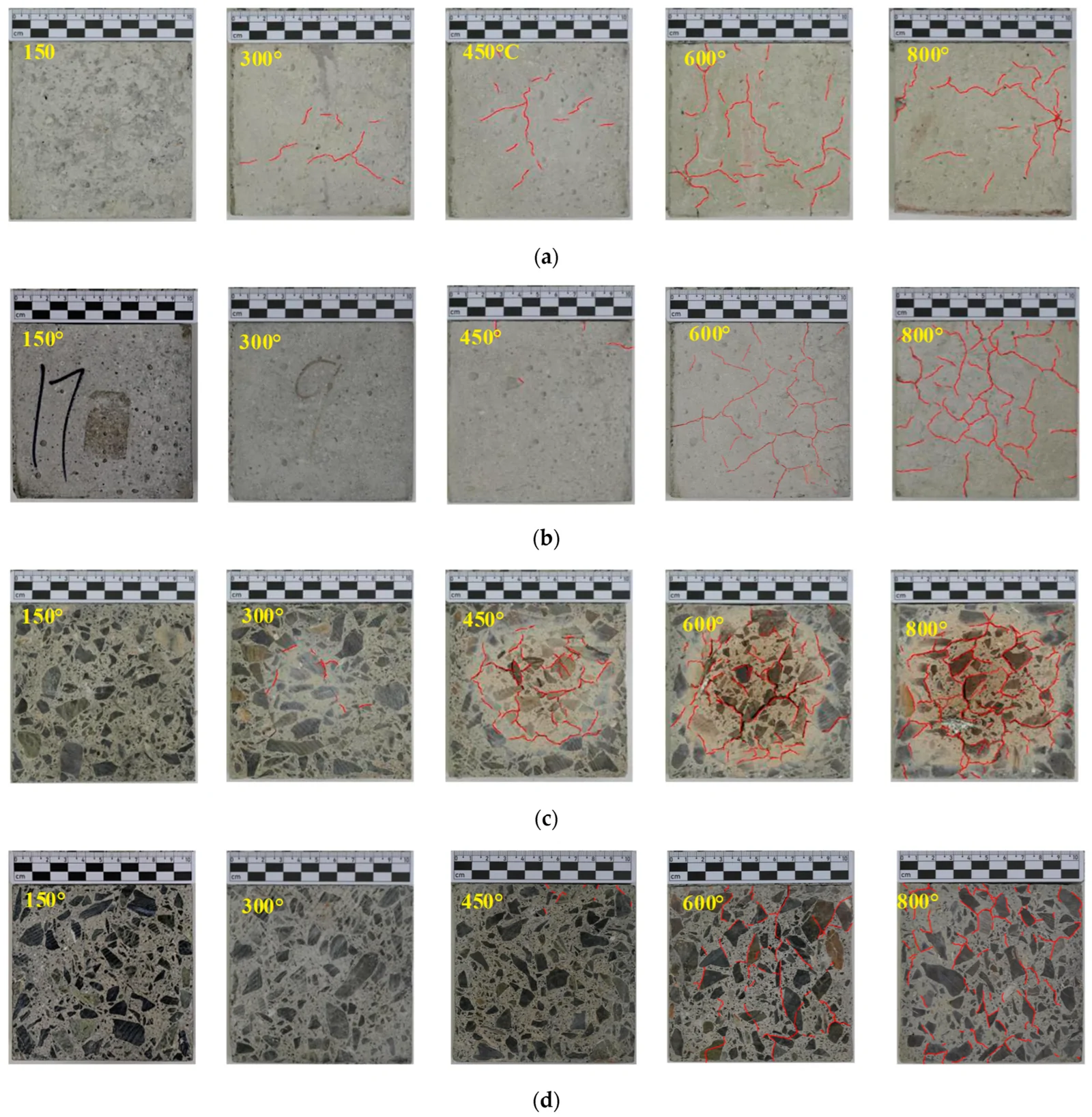
Variation in macroscopic morphology of the specimens with temperature (comparison between MW and MF groups). (**a**) External surface of MW; (**b**) external surface of MF; (**c**) internal cross-section of MW; (**d**) internal cross-section of MF.

**Figure 7:**
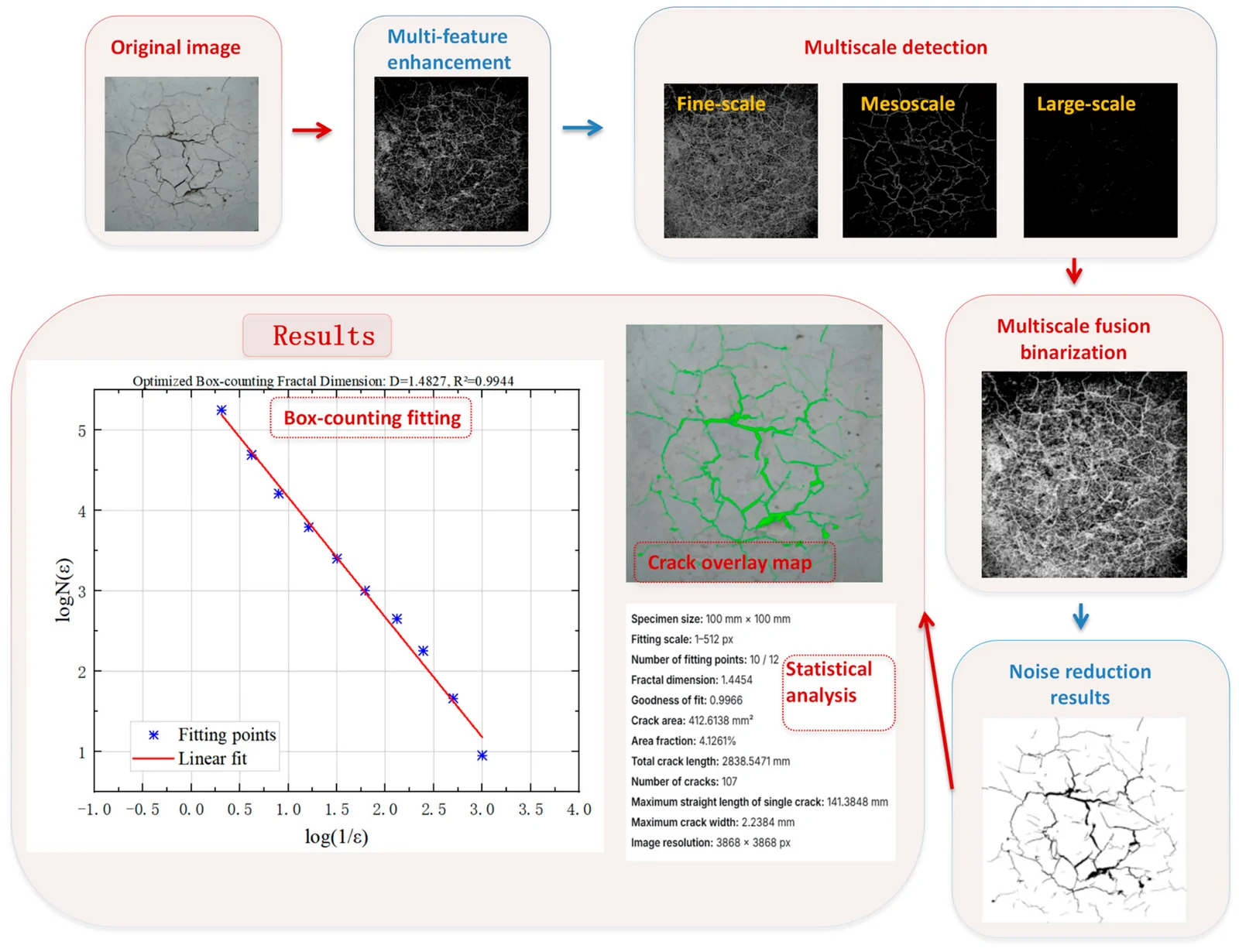
Complete Process of Typical Crack Identification (MW Group, 450 °C Cross-Section).

**Figure 8:**
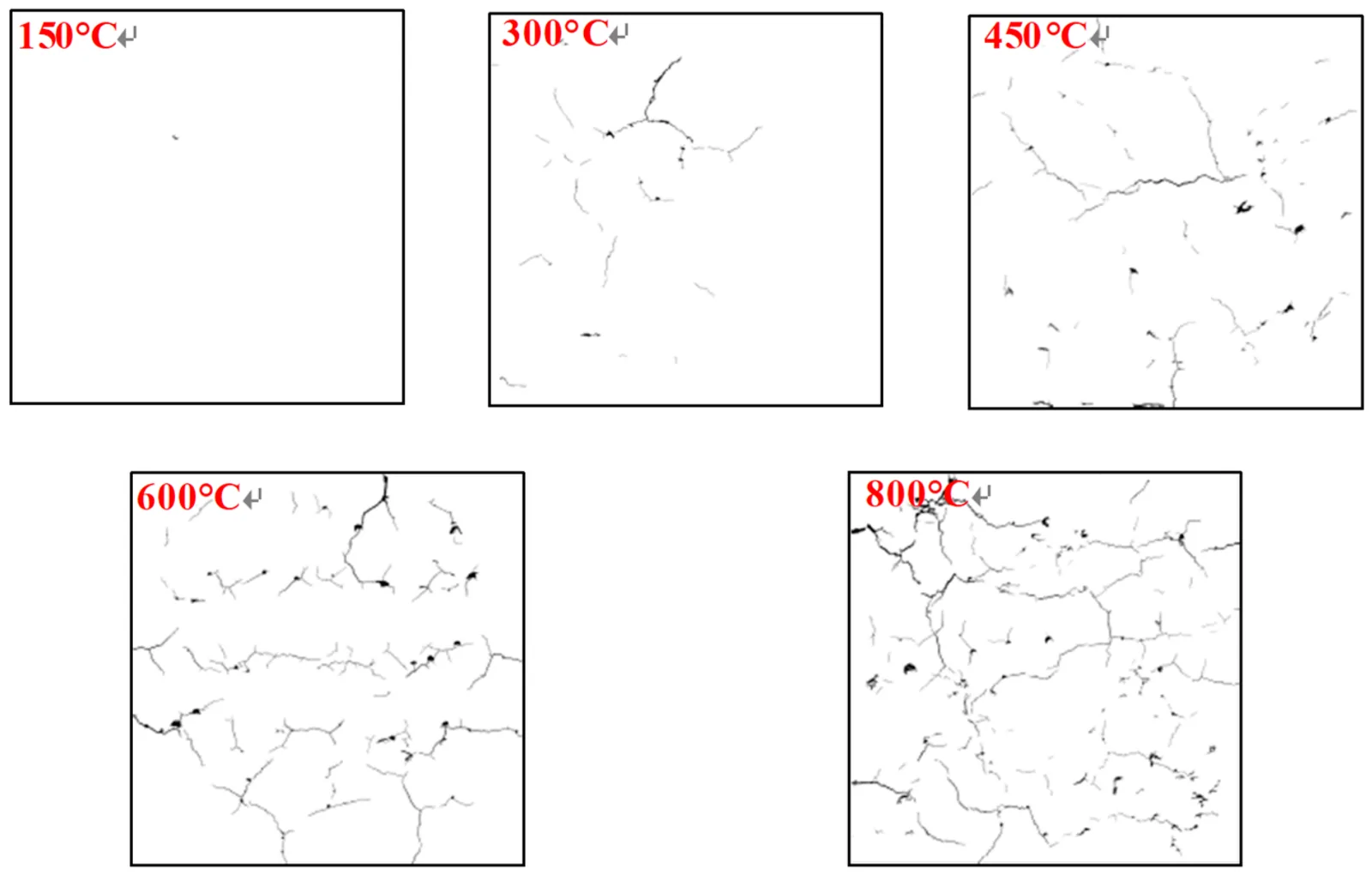
Binarized images of the external surfaces of the MW group (150~800 °C).

**Figure 9:**
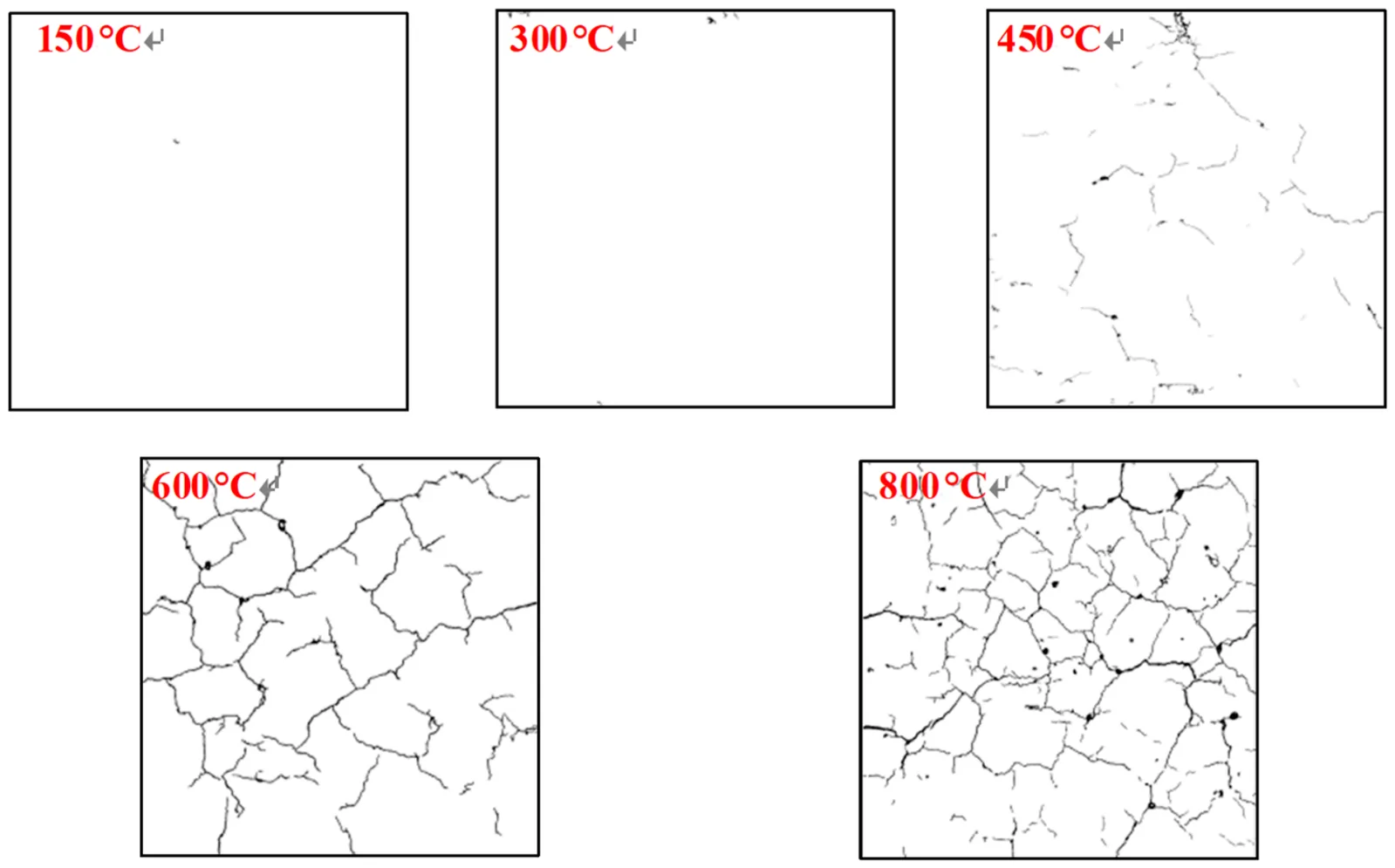
Binarized images of external surfaces of the MF group (150~800 °C).

**Figure 10:**
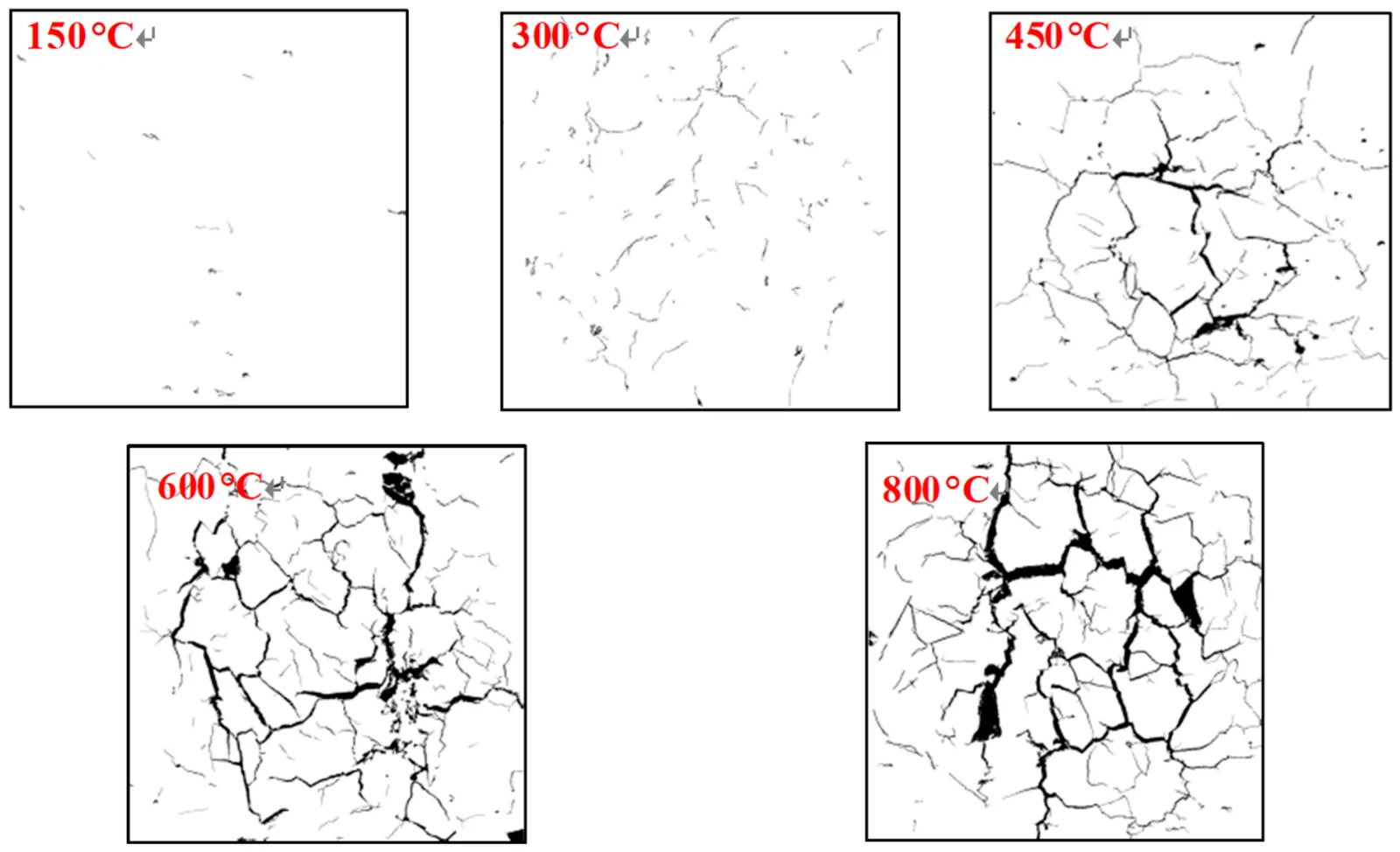
Binarized images of the internal cross-sections of the MW group (150~800 °C).

**Figure 11:**
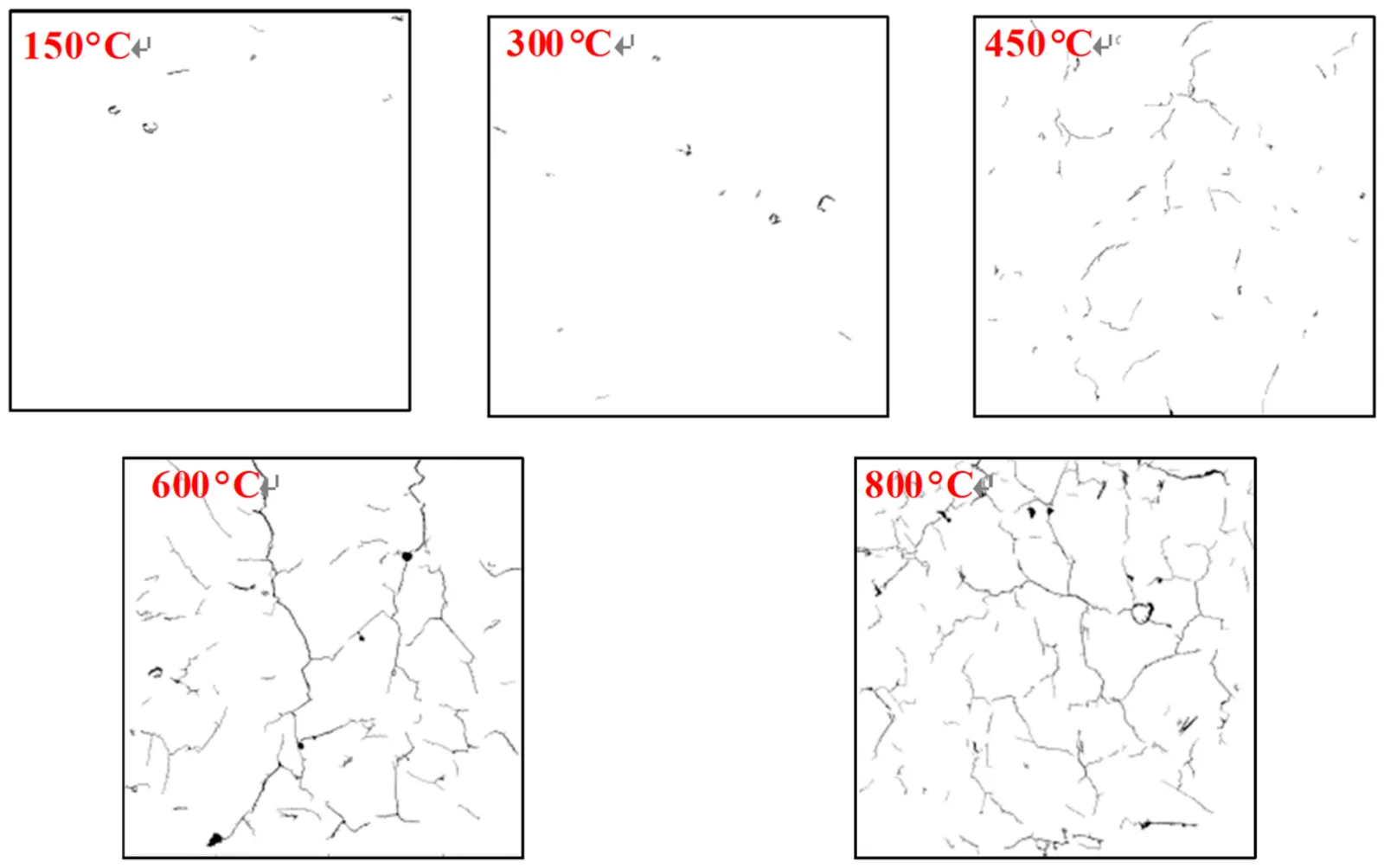
Binarized images of the internal cross-sections of the MF group (150~800 °C).

**Figure 12:**
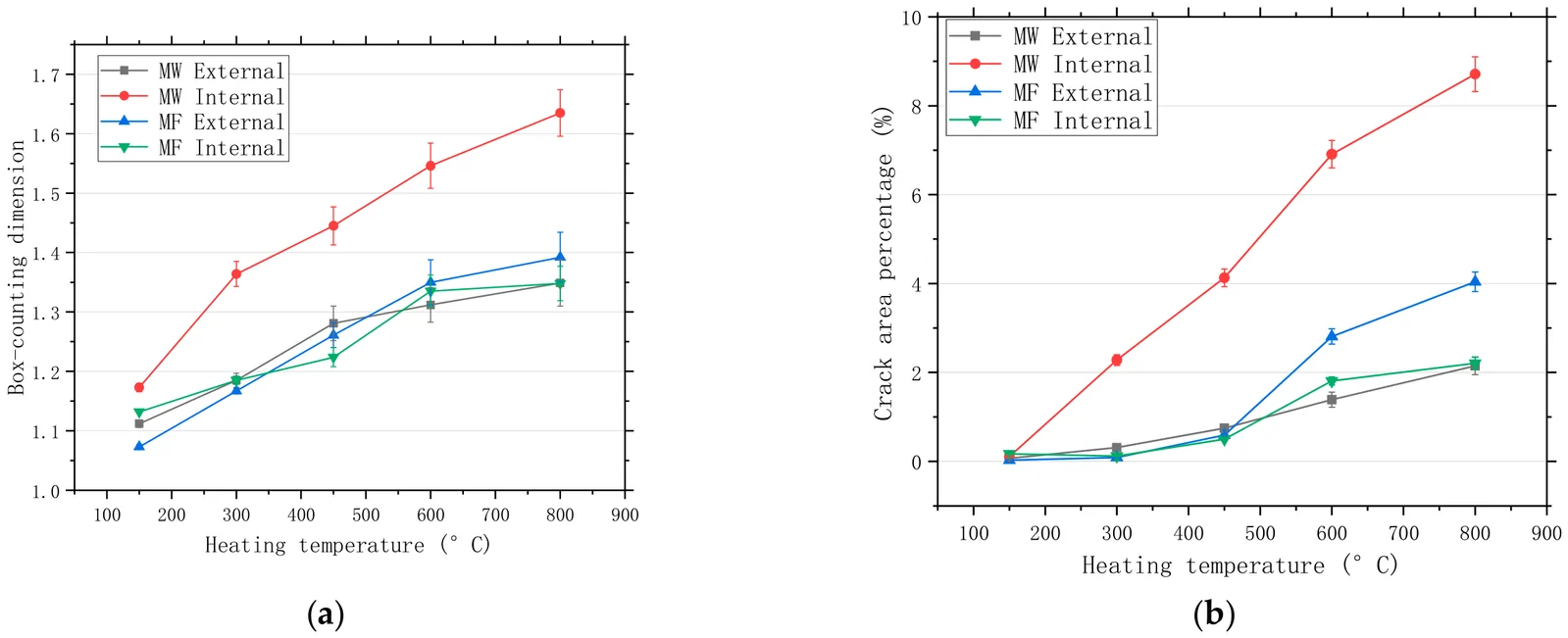
Quantitative crack characteristics. (**a**) fractal dimension $D_{b}$; (**b**) crack area percentage.

**Figure 13:**
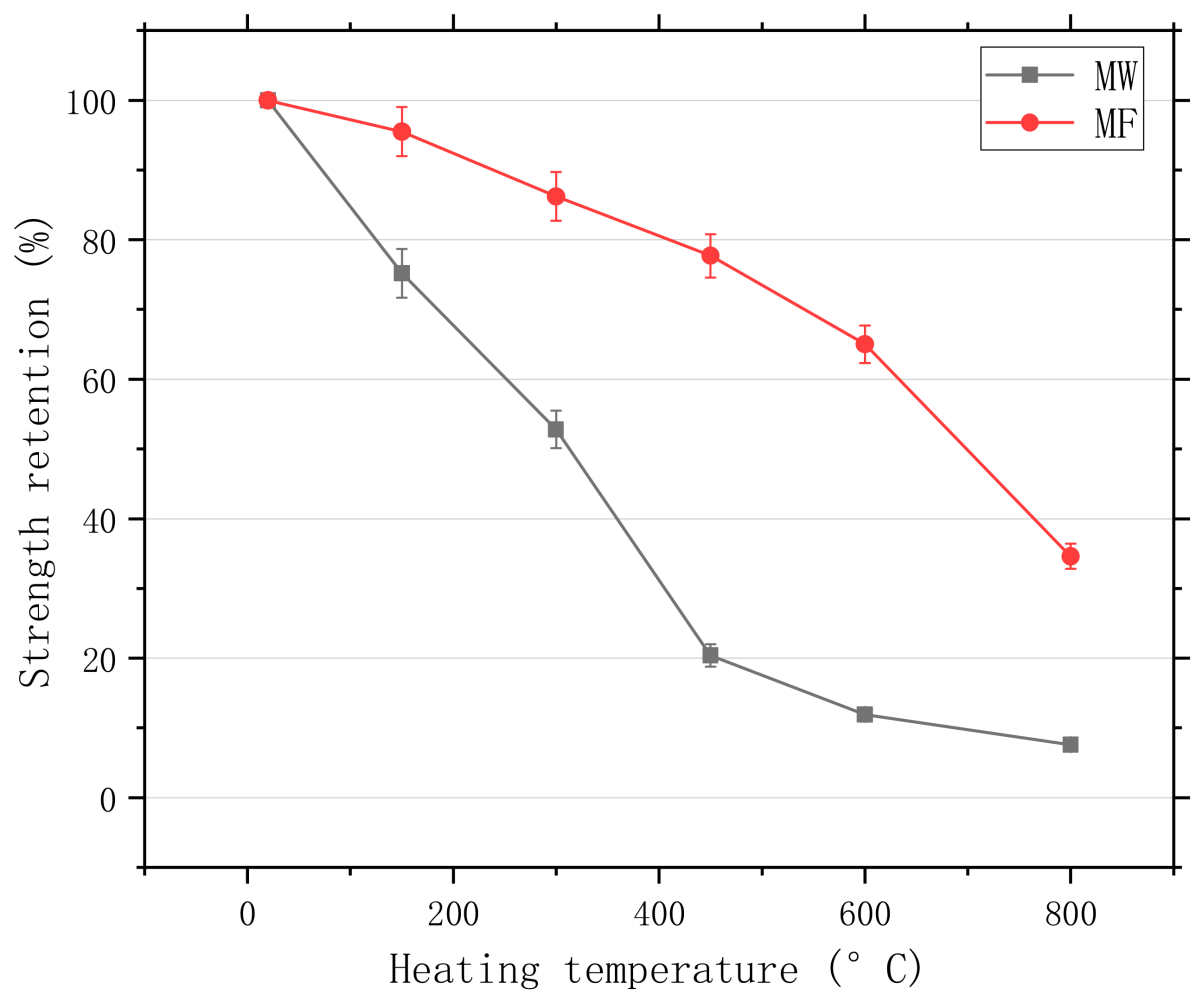
Curve showing the strength retention rate as a function of heating temperature.

**Figure 14:**
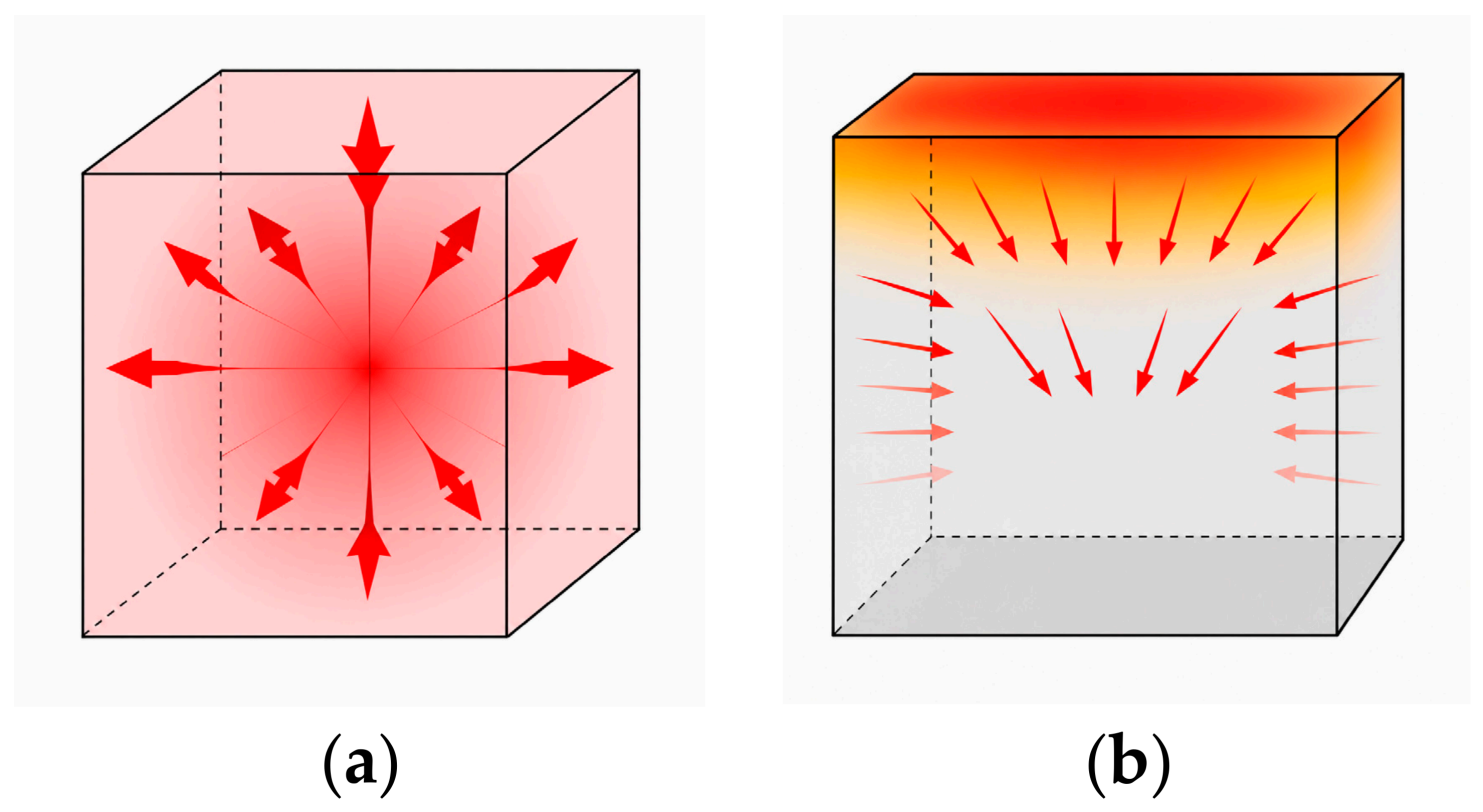
Schematic illustration of heating modes and temperature gradient directions. (**a**) MW volumetric heating (inside-out gradient); (**b**) MF surface heating (top-down gradient).

**Figure 15:**
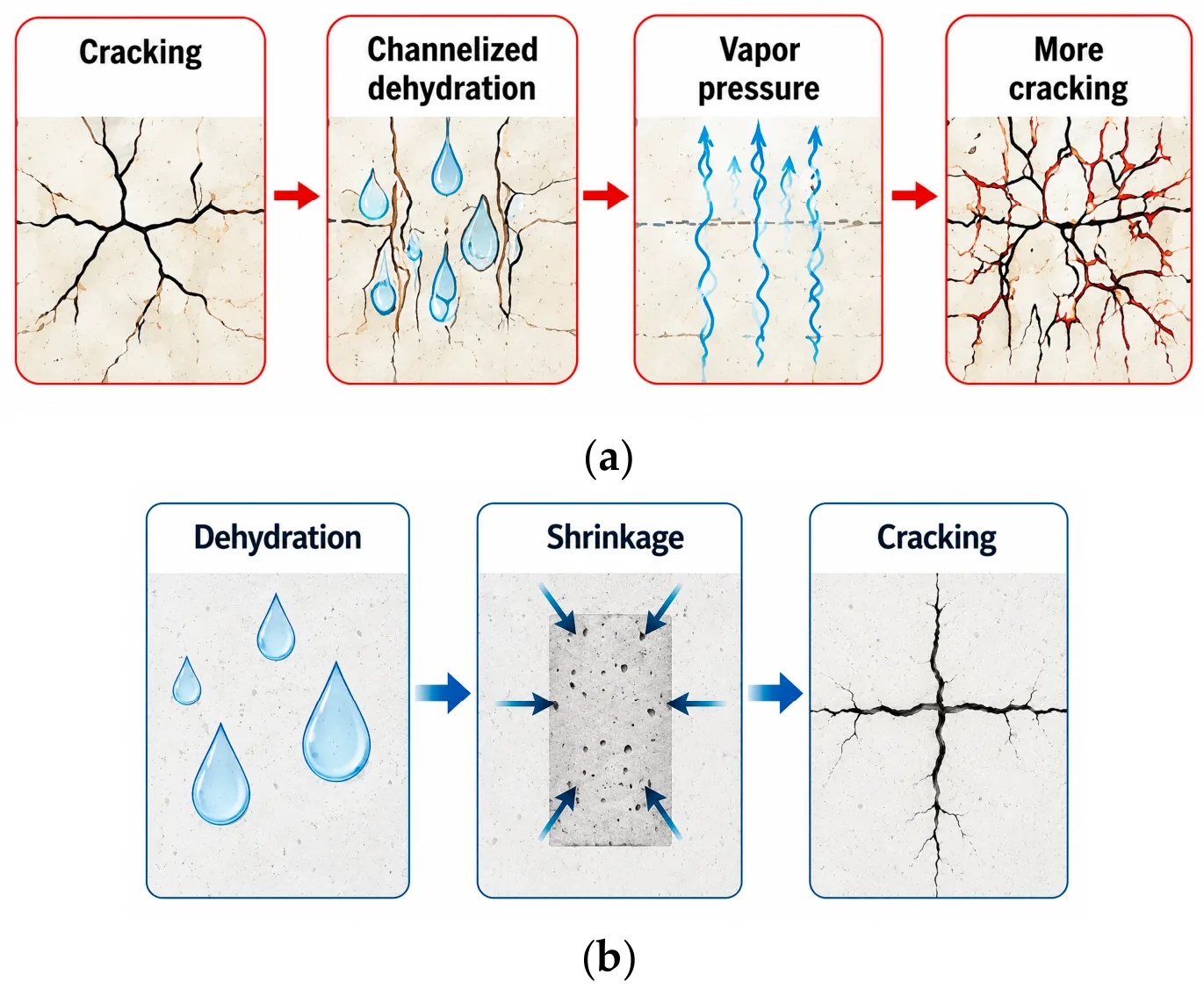
Schematic illustration of damage mechanisms. (**a**) positive feedback loop between dehydration and cracking in the MW group; (**b**) sequential dehydration–shrinkage–cracking mechanism in the MF group.

**Figure 16:**
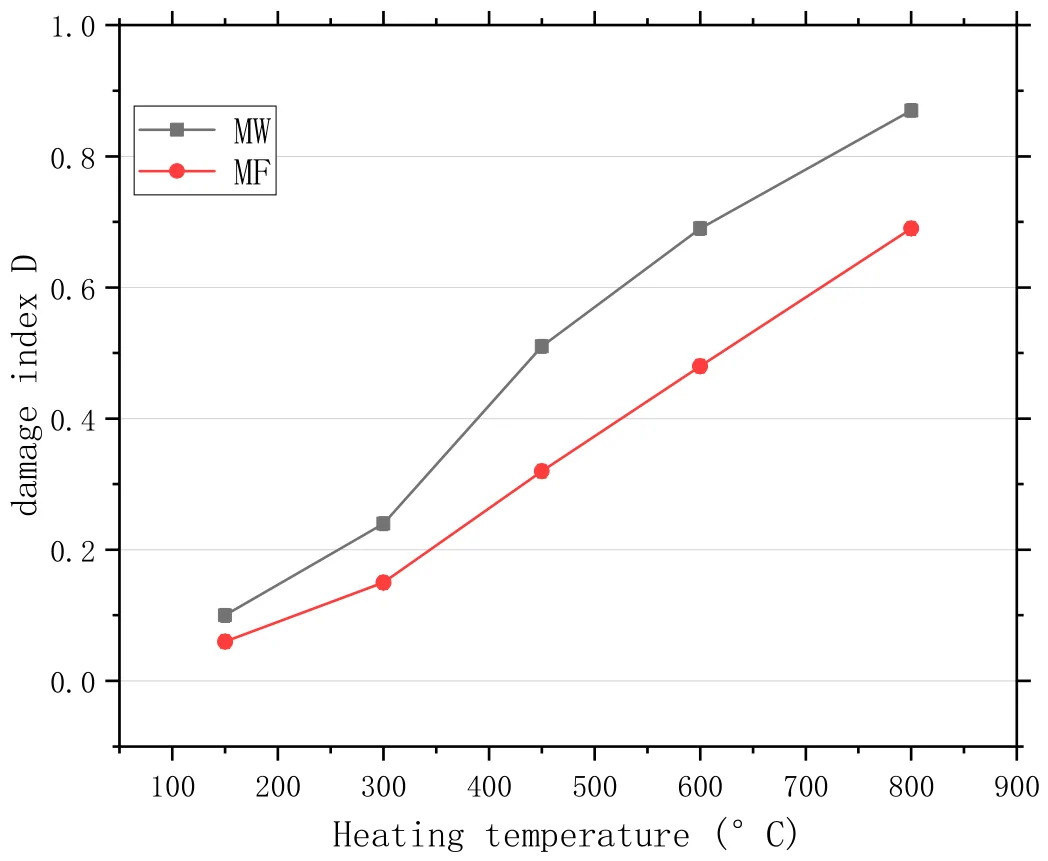
Comprehensive damage index *D* as a function of heating temperature.

**Table 1:** Major chemical composition of serpentine aggregate (wt.%).

Oxide	MgO	SiO_2_	Al_2_O_3_	Fe_2_O_3_	CaO	K_2_O	NiO	Cr_2_O_3_
Content (wt.%)	42.75	40.28	1.89	2.07	1.40	1.20	0.45	1.46

**Table 2:** Specimen grouping and test programme.

Specimen Type	Number of Specimens	Heating Method	Target Temperatures (°C)	Replicates	Measured Parameters
Intact specimens	50	MW/ MF	150, 300, 450, 600, 800	5	Mass loss, residual compressive strength, and surface temperature distribution (in situ)
Sectioned specimens	50	MW/ MF	150, 300, 450, 600, 800	5	Internal temperature distribution (in situ) and crack characteristics (after cooling)
Room-temperature control group	5	-	-	5	Initial compressive strength
Total	105	-	-	-	-

**Table 3:** Temperature Differences and Thermal Values (TV) at Different Temperature Points.

Heating Temperature (°C)	Group	External Surface					Internal Cross-Section				
		Max. (°C)	Min. (°C)	$\Delta T_{S}$ (°C)	Avg. (°C)	*TV*	Max. (°C)	Min. (°C)	$\Delta T_{I}$(°C)	Avg. (°C)	*TV*
150	MW	433.7	147.2	286.5	362.1	7.91	563.3	147	416.3	449.8	9.26
	MF	156.0	114.1	41.9	130.3	3.22	148.6	94.3	44.3	117.8	3.76
300	MW	538.5	304.4	234.1	451	5.19	752.1	298	454.1	611.6	7.42
	MF	302.8	239.3	63.5	257.2	2.47	293.3	222.7	70.6	248.3	2.85
450	MW	694.0	449.3	244.7	594.8	4.11	925.7	451.3	474.5	701.3	6.77
	MF	450.0	364.5	85.4	402.8	2.12	446.2	349.4	96.8	386.5	2.50
600	MW	768.9	597.2	171.7	678.7	2.53	1128.8	601.7	527.1	932.2	5.65
	MF	606.1	523.9	82.2	567.5	1.45	600.7	490.0	110.7	561.7	1.97
800	MW	857.4	787.4	70.0	817.6	0.86	1211.0	785.2	425.8	1051.7	4.05
	MF	800.6	702.3	98.2	763.1	1.29	802.8	704.3	98.6	744.3	1.32

Note: Temperature fields were acquired from five replicate specimens. The values reported here are from one representative specimen. The coefficient of variation in the maximum surface and internal temperatures among the five replicates was below 3.5% for all conditions. The data in Table 3 are also presented graphically in Figure 4.

**Table 4:** Mass Loss Rate (%).

Heating Temperature (°C)	Max. (°C)	Min. (°C)	MW			MF			Difference
			Mass Before Heating (g)	Mass After Heating (g)	Mass Loss Rate (%)	Mass Before Heating (g)	Mass After Heating (g)	Mass Loss Rate (%)	
150	563.3	94.3	2216	2153	2.84 ± 0.09	2239	2211	1.25 ± 0.06	1.55
300	752.1	222.7	2219	2108	5.00 ± 0.13	2250	2187	2.80 ± 0.10	2.20
450	925.7	349.4	2277	1994	12.43 ± 0.24	2240	2126	5.09 ± 0.19	7.31
600	1128.8	490	2259	1940	14.12 ± 0.23	2298	2112	8.09 ± 0.25	6.08
800	1211	704.3	2161	1807	16.38 ± 0.31	2219	1910	13.93 ± 0.30	2.45

Note: Mass loss rate is reported as mean ± standard deviation of five replicate specimens (n = 5).

**Table 5:** Quantitative Statistics of Crack Characteristics (Box-counting method).

Heating Temperature (°C)	Group	External Surface				Internal Cross-Section			
		Box-Counting Dimension	Max Length (mm)	Max Width (mm)	Crack Area Percentage (%)	Box-Counting Dimension	Max Length (mm)	Max Width (mm)	Crack Area Percentage (%)
150	MW	1.112 ± 0.006	3.23	0.25	0.07 ± 0.01	1.173 ± 0.007	5.32	0.45	0.11 ± 0.01
	MF	1.073 ± 0.004	1.76	0.22	0.03 ± 0.00	1.132 ± 0.002	3.44	0.51	0.17 ± 0.01
300	MW	1.185 ± 0.012	21.4	0.61	0.31 ± 0.03	1.364 ± 0.021	36.4	1.98	2.28 ± 0.12
	MF	1.167 ± 0.005	5.41	0.55	0.09 ± 0.01	1.185 ± 0.007	4.31	0.57	0.12 ± 0.01
450	MW	1.281 ± 0.029	35.22	1.13	0.75 ± 0.09	1.445 ± 0.032	45.17	2.24	4.13 ± 0.20
	MF	1.261 ± 0.021	14.99	0.79	0.59 ± 0.03	1.224 ± 0.016	15.9	0.92	0.50 ± 0.03
600	MW	1.312 ± 0.029	35.14	1.22	1.39 ± 0.17	1.546 ± 0.038	77.89	4.71	6.91 ± 0.31
	MF	1.350 ± 0.038	77.01	1.23	2.81 ± 0.17	1.335 ± 0.027	47.89	1.39	1.81 ± 0.09
800	MW	1.349 ± 0.039	57.94	1.34	2.15 ± 0.20	1.635 ± 0.039	98.44	4.93	8.71 ± 0.39
	MF	1.392 ± 0.042	99.90	1.61	4.04 ± 0.22	1.348 ± 0.029	42.73	2.14	2.21 ± 0.14

Note: Box-counting dimension and crack area percentage are reported as mean ± standard deviation of five replicate specimens (n = 5). Max length and max width are the maximum values observed among the five specimens.

**Table 6:** Residual compressive strength and retention rate.

Heating Temperature (°C)	MW Strength (Mpa)	MW Strength Retention (%)	MF Strength (MPa)	MF Strength Retention (%)
Room temperature	31.15 ± 0.72	100	31.15 ± 0.72	100
150	23.45 ± 0.93	75.2 ± 3.5	29.67 ± 0.84	95.5 ± 3.5
300	16.41 ± 0.75	52.8 ± 2.7	27.37 ± 0.90	86.2 ± 3.5
450	6.35 ± 0.47	20.4 ± 1.6	24.15 ± 0.79	77.7 ± 3.1
600	3.69 ± 0.30	11.9 ± 1.0	20.19 ± 0.70	65.0 ± 2.7
800	2.36 ± 0.28	7.6 ± 0.9	10.78 ± 0.49	34.6 ± 1.8

Note: MW and MF strength and strength retention are reported as mean ± standard deviation of five replicate specimens (n = 5).

**Table 7:** Comparison of internal temperature differences and dehydration rates.

Heating Temperature (°C)	MW Internal $\Delta T_{I}$ (°C)	MF Internal $\Delta T_{I}$ (°C)	$\Delta T_{I}$ Ratio (%)	MW Mass Loss (%)	MF Mass Loss (%)
300	454.1	70.6	6.4	—	—
450	474.5	96.8	4.9	7.40	2.29
600	527.1	110.7	4.8	—	—

**Table 8:** Evolution of internal temperature differences and crack area in the MW group.

Heating Temperature (°C)	$\Delta T_{I}$ (°C)	Area of Internal Cracks (%)
300	454.1	2.19
450	474.5	3.31
600	527.1	6.91
800	425.8	7.74

**Table 9:** Correction factors for heating mechanisms.

Heating temperature (°C)	150	300	450	600	800
κ	0.787	0.613	0.269	0.232	0.220

**Table 10:** Comprehensive damage index *D*.

Heating Temperature (°C)	MW Group	MF Group
150	0.10 ± 0.01	0.06 ± 0.01
300	0.24 ± 0.01	0.15 ± 0.01
450	0.51 ± 0.01	0.32 ± 0.01
600	0.69 ± 0.01	0.48 ± 0.01
800	0.87 ± 0.01	0.69 ± 0.01

Note: Values are mean ± standard deviation of five replicate specimens (n = 5). The standard deviation was propagated from Table 4, Table 5 and Table 6. The propagated uncertainty was approximately 0.01 for all conditions.

**Table 11:** Classification criteria for injury severity.

Damage Level	*D*-Value	Condition Description	Engineering Recommendations
Minor damage	D<0.2	No visible cracks; structure largely intact	Normal operation
Mild damage	0.2≤D<0.4	Initial microcrack	Increased inspection
Moderate damage	0.4≤D<0.6	Noticeable crack propagation	Assessment and repair recommended
Severe damage	0.6≤D<0.8	Through-cracks formed	Restricted use
Serious damage	D≥0.8	Severe structural deterioration	Replace immediately

**Table 12:** Engineering Criteria Framework.

Criterion	Critical Value	Physical Basis	Engineering Application
Direction of temperature gradient	MW: Inside to outside; MF: Top to bottom	Thermoelastic theory	Qualitative assessment of likely damage patterns
Internal $\Delta T_{i}$	MW gradient significantly larger than MF	Dehydration rate + Crack propagation	Assess severity of damage evolution
Internal vs. external crack area ratio	MW: inside > outside; MF: outside > inside	Crack development patterns	Identify primary damage pathways
Crack area threshold	Internal cross-sectional crack area > 5%	The relationship between crack area and strength	Early warning of notable strength loss
$D_{b}$	$D_{b}>1.5$	Crack network formation	Indicates potential sudden strength drop
κ(T)	κ=0.25 (T ≥ 450 °C)	Strength Retention Ratio	Adjust predictions for volumetric heating
Comprehensive damage *D*	*D* ≥ 0.6	Multi-parameter Fusion	Classification of structural safety conditions
Temperature offset	~200 °C in advance	*D*-value Comparison	Reference for Design Temperature Margin

## Data Availability

The original contributions presented in this study are included in the article. Further inquiries can be directed to the corresponding authors.
